# Automatically pre-screening patients for the rare disease aromatic l-amino acid decarboxylase deficiency using knowledge engineering, natural language processing, and machine learning on a large EHR population

**DOI:** 10.1093/jamia/ocad244

**Published:** 2023-12-22

**Authors:** Aaron M Cohen, Jolie Kaner, Ryan Miller, Jeffrey W Kopesky, William Hersh

**Affiliations:** Department of Medical Informatics and Clinical Epidemiology, School of Medicine, Oregon Health & Science University, Portland, OR 97239, United States; Department of Medical Informatics and Clinical Epidemiology, School of Medicine, Oregon Health & Science University, Portland, OR 97239, United States; PTC Therapeutics, South Plainfield, NJ 07080, United States; PTC Therapeutics, South Plainfield, NJ 07080, United States; Department of Medical Informatics and Clinical Epidemiology, School of Medicine, Oregon Health & Science University, Portland, OR 97239, United States

**Keywords:** machine learning, rare diseases, natural language processing, EHR data secondary uses, aromatic l-amino acid decarboxylase deficiency

## Abstract

**Objectives:**

Electronic health record (EHR) data may facilitate the identification of rare diseases in patients, such as aromatic l-amino acid decarboxylase deficiency (AADCd), an autosomal recessive disease caused by pathogenic variants in the dopa decarboxylase gene. Deficiency of the AADC enzyme results in combined severe reductions in monoamine neurotransmitters: dopamine, serotonin, epinephrine, and norepinephrine. This leads to widespread neurological complications affecting motor, behavioral, and autonomic function. The goal of this study was to use EHR data to identify previously undiagnosed patients who may have AADCd without available training cases for the disease.

**Materials and Methods:**

A multiple symptom and related disease annotated dataset was created and used to train individual concept classifiers on annotated sentence data. A multistep algorithm was then used to combine concept predictions into a single patient rank value.

**Results:**

Using an 8000-patient dataset that the algorithms had not seen before ranking, the top and bottom 200 ranked patients were manually reviewed for clinical indications of performing an AADCd diagnostic screening test. The top-ranked patients were 22.5% positively assessed for diagnostic screening, with 0% for the bottom-ranked patients. This result is statistically significant at *P* < .0001.

**Conclusion:**

This work validates the approach that large-scale rare-disease screening can be accomplished by combining predictions for relevant individual symptoms and related conditions which are much more common and for which training data is easier to create.

## Background and significance

Aromatic l-amino acid decarboxylase deficiency (AADCd) is an autosomal recessive disorder caused by pathogenic variants in the dopa decarboxylase (*DDC*) gene. This gene encodes the AADC enzyme, which is responsible for catalyzing the chemical reactions that create the neurotransmitters: epinephrine, norepinephrine, dopamine, and serotonin. Therefore, the deficiency has widespread neurological effects including hypotonia, movement disorders such as oculogyric crisis and dystonia, dysfunction of the autonomic nervous system, and developmental delay.^1^

As is the case with many rare disorders, estimating the prevalence of AADCd is challenging. The true global prevalence is unknown. In the most comprehensive recent study, 348 cases have been described worldwide, with a higher prevalence in Taiwan due to a founder variant.^2^ Most patients present in infancy with hypotonia, oculogyric crisis, developmental delay, and feeding issues. Patients with the classic form of AADCd never reach their gross motor developmental milestones. Sleep disorders, gastrointestinal (GI) problems, mood disturbance, and feeding issues are frequent. AADCd has a wide spectrum of phenotypes with cases presenting late in childhood or early in adulthood and remaining undetected, perhaps indefinitely.^3^

AADCd often presents as a nonspecific neurodevelopmental disorder, particularly when the distinguishing feature of the oculogyric crisis is not recognized. As a result, patients often receive other clinical diagnoses, such as cerebral palsy and/or seizures, prior to AADCd being recognized as the underlying etiology.^4–8^ Furthermore, the primary diagnostic methodology of CSF neurotransmitter analysis may be underutilized due to the invasiveness of lumbar puncture and the limited availability of the analysis (we are aware of only 2 clinical laboratories providing the testing in the United States). These factors likely lead to AADCd being underdiagnosed.^1^^,^^9^

According to consensus guidelines, a definitive diagnosis should include positive findings in 2 of the 3 core diagnostic tests^10^:

1. cerebrospinal fluid analysis demonstrating abnormal levels of neurotransmitter metabolites consistent with deficiency of the AADC enzyme;
2. reduced plasma AADC enzyme activity;
3. compound heterozygous or homozygous pathogenic variants in the *DDC* gene.

Other biochemical tests which can support a diagnosis of AADCd include measurement of 3-O-methyldopa (3-OMD) in dried blood spots or plasma, or urine organic acid analysis.^2^

Treatment with dopamine agonists, MAO inhibitors, and pyridoxine/pyridoxal phosphate has shown limited efficacy in some AADCd patients,^10^ and gene therapy treatments are currently approved in Europe and the United Kingdom, and in development in the United States and China. Therefore, a systematic approach to identifying patients at an increased risk of AADCd is warranted.

There are as many as 10 000 rare diseases around the world.^11^ The time to diagnosis for many of these diseases is lengthened by their rarity as well as under-recognition by evaluating clinicians. This may cause needless suffering for such patients, not only in the stress of not having a diagnosis for their symptoms but also, when they exist, delays in treatment to reduce the symptoms of these diseases, which are sometimes debilitating. One possible way to expedite the diagnosis of rare diseases is through the use of clinical data, particularly data in the electronic health record (EHR)^12^ coupled with new advances in machine learning (ML).^13^ Many patients with rare diseases see numerous providers, resulting in a corpus of data that can be processed to uncover signals of rare diseases.

Our previous work focused on acute hepatic porphyria (AHP), a rare disease occurring in approximately 1 per 100 000 people.^14^ The time to diagnosis of AHP takes an average of 15 years from the onset of symptoms.^15^ Our previous work on a corpus of EHR data from 205 000 patients, with 30 positive cases, found that we could identify the presence of the neuro-visceral symptoms of AHP and no other explanatory diagnoses using a ML approach.^16^ While we were not able to diagnose any new cases from 7 of 18 people who our algorithm identified and were willing to undergo urine porphobilinogen testing, it was clinically appropriate to test such individuals.^17^

Others have searched for additional rare diseases in EHR data using ML. These include births of patients with cardiac amyloidosis,^18^ systemic sclerosis,^19^ lipodystrophy,^20^ presence of the KCNA2 gene variant,^21^ primary Sjögren’s syndrome,^22^ Dravet syndrome,^23^ Jeune syndrome,^24^ systematic lupus erythematosus,^25^ renal ciliopathies,^26^ Pompe disease,^27^ and Fabry disease.^28^

## Objectives

The goal of this study was to use EHR data to identify patients who may have undiagnosed AADCd and possibly other related disorders of aromatic amino acid and neurotransmitter metabolism that may be coded similarly in the EHR (eg, ICD10 coding E70.81). Patients who have AADCd, but are yet to be diagnosed, will of course not have a structured diagnostic code in the EHR for this disease. In the initial review of 500K patients aged ≤ 25 years old in the Oregon Health & Science University (OHSU) Research Data Warehouse (RDW), one patient was found with this diagnostic code assigned. Clearly, this is a very rare disease, and is potentially underrecognized, even in a tertiary care facility such as OHSU, which is the largest academic medical center in Oregon.

EHR data provide a wealth of information to improve clinical care and facilitate research. Identifying patients for further diagnostic work up of undiagnosed rare diseases by manual chart review is a laborious, time-consuming, and likely impractical task. This research was the first attempt to develop and evaluate an algorithm to identify potential patients with this disease on a large and realistic EHR dataset.

This approach could facilitate more accurate population prevalence assessment as well as provide proof of concept for a tool that can help identify undiagnosed patients who may benefit from earlier treatment as well as eligibility for future clinical trials. As opposed to more traditional projects of this type that rely entirely on manual chart review to identify potential patients, our approach made use of informatics, information retrieval, natural language processing (NLP), and ML techniques to create a more efficient and reusable approach to identifying patients who potentially have AADCd. While prior work exists on detecting cases of rare diseases in EHR data (see Background and Significance), this was the first work on AADCd and the first proposed method that we are aware of using ML and which did not use or have sample cases of the rare disease for training or algorithmic tuning.

## Methods

### Overall approach based on symptoms, not disease cases

**Figure 1.**
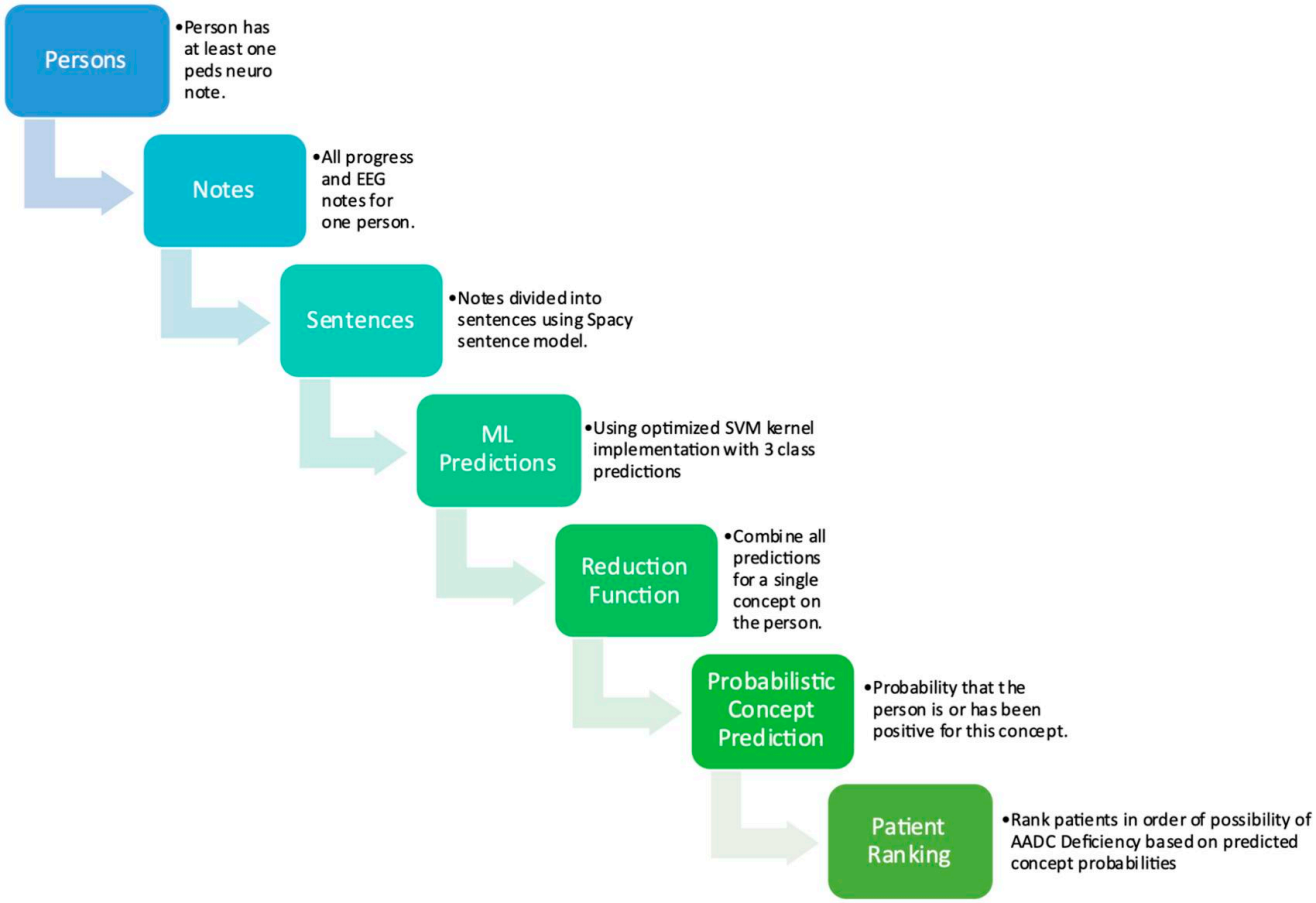
Sentence to patient-ranking process. The overall process, starting with an identified cohort of potential patients, processing notes into sentences, scoring sentences, combining scores, and ranking patients is shown in this flowchart.

The overall strategy for the automated identification of patients who may have undiagnosed AADCd and should be considered for diagnostic screening is shown in Figure 1.

This approach is not based on direct training with positive AADCd cases. Instead, the proposed method is based on recognizing the symptoms and associated conditions that together may indicate an undiagnosed rare disorder of neurotransmitter metabolism. Specifically, the goal is to identify cases where a definitive diagnosis is not present in the chart and diagnostic testing for AADCd may be indicated.

The approach follows a multistep process:

1. Based on the literature review and expert knowledge, a set of AADCd-associated symptoms and conditions of interest is identified.
2. Randomly divide the dataset of patient chart data meeting inclusion criteria (described below) into 10 partitions. Use partition 0 for development and testing. Hold out partitions 1-9 as the blinded dataset.
3. Divide clinical note data for partition 0 into training 80%, validation 10%, and testing 10%, by randomly assigning patients to 1 of the 3 sets.
4. Create an annotation guide and manually annotate a training set of Pediatric Neurology and EEG exam notes annotated for the associated symptoms and conditions of interest.
5. Divide patient pediatric neurology and EEG exam notes into sentences.
6. Score each sentence for the probability of containing an AADCd concept of interest using a trained ML model
7. Combine all sentence predictions for an individual patient into a single probabilistic prediction of whether the patient had expressed that AADCd concept in a positive manner in a note.
8. Combine all individual concept predictions into a single rank value using a fitted Poisson regression model.
9. Manually review the top-ranked patient charts for diagnostic testing consideration. Also manually review the bottom-ranked patient charts for comparison.

#### Creation of annotation schema

After a review of the literature^3^^,^^5^^,^^10^^,^^29–32^ and discussion amongst all authors, it was determined that the following 10 concepts were the most important factors in determining the risk of AADCd from the EHR:

1. Autonomic dysfunction
2. Cerebral palsy
3. Developmental delay
4. Epilepsy or seizures
5. Feeding issues
6. Hypotonia
7. Insomnia
8. Mood disturbances
9. Movement disorders
10. Oculogyric crisis

These concepts of interest are a combination of symptoms related to AADCd, and conditions that may co-occur or be differential diagnoses for AADCd. The concepts of interest were also annotated with modifiers designating whether they were negated, not about the patient, or hypothetical. The listed concepts are not all of the same diagnostic or screening importance and are specified in alphabetical order and not ranked order.

For completeness, we also annotated whether the patient chart specifically mentioned AADCd. This was a very rare occurrence in our dataset, and we did not use direct mention of AADCd as a concept in our ML models. The approach is geared toward identifying patients with unrecognized AADCd, and it was reasoned that if AADCd is mentioned in the chart, it is already being considered by the clinicians, and therefore identifying these patients automatically would not add value as far as suggesting diagnostic testing.

#### Creation of pediatric neurology focused dataset

For all data in this study, the Observational Medical Outcomes Partnership (OMOP) Common Data Model (CDM)^33–35^ research database instance was provided by the OHSU Research Data Warehouse (RDW). This research data source includes all OHSU patients represented in a standards-based data model. By basing our work on this data model, the results are intended to be more generalizable and reusable. This study was approved by the OHSU IRB under approval number STUDY00023368.

The initial dataset cohort was generated by creating a subset of the main OMOP database by requiring patients to be ≤25 years old and have at least 2 visits at OHSU. A text search of the OMOP NOTES database table for this age criteria cohort dataset was then performed, identifying patients who had at least one Pediatric Neurology note. Using this process, the study cohort dataset was created, which consisted of 8946 patients who met the following set of selection criteria and had sufficient data to be included in this study:

- ≤25 years of age at the time of the data set creation
- ≥2 visits in the OHSU OMOP database
- ≥1 Pediatric Neurology note

The study cohort dataset was then divided into 10 partitions of approximately 850 patients each. Partition 0 was used for all investigation and training. Partitions 1-9 were used later in the study as test data to test the application of the approach on unseen data.

After reviewing a random selection of notes in partition 0, it was determined that most of the notes did not reference or contain much information that was relevant to the detection of AADCd. To focus the dataset on information relevant to AADCd detection, the notes were further filtered by limiting the final experimental dataset to Pediatric Neurology notes and EEG reports. For this study, other note types were not processed or manually reviewed.

The resulting experimental dataset contained 8946 patients and 520 473 notes overall. Partition 0 contained 921 patients and 54 857 notes.

#### Annotation of partition 0

To create a training data set for the AADCd-related concepts, an annotator trained in epidemiology (J.K.) reviewed the notes for each patient in partition 0 and selected the most clinically complete appearing early and late pediatric neurology note in the record, as well as the most complete EEG. This was done to maximize the efficiency of manual annotation to get as much data on the concepts of interest from the annotated notes.

The BRAT^36^ annotation tool was installed locally and used by the annotator to select text spans and save annotations. For each of the selected notes, the annotator selected the minimum span of text that expressed the complete concept in the annotation schema, including any modifiers. The annotation schema allowed overlap of annotations, if necessary, such as a single negation applying multiple annotated concepts.

An initial round of 20 patients was first annotated, and these annotations were reviewed by the PI (A.M.C.). After a discussion of how to handle some uncertain edge cases and discovering some inconsistencies, the annotation guide was enhanced to provide additional specific instructions and example phrases. After updating and reviewing the annotation guide, the rest of the selected notes in partition zero were annotated. The final annotation guide is available as Appendix SA1.

#### Creation of training, validation, and test datasets

The annotated partition 0 data were converted into a training dataset suitable for ML by a multistep process. The goal of this process was to create a set of sentences, each sentence having an associated binary variable designating whether or not the sentence included the AADC-related concept, and other associated binary variables for each concept about whether they were negated, not about the patient, or hypothetical.

Each note was parsed into individual sentences using the “en_core_web_trf” model sentence parser in the spacy (https://spacy.io) Python toolkit. This model was the most complex parsing model and was chosen as having the best documented performance. Custom Python scripts were then written, which used sentence offset and text matching to determine which sentences corresponded to the individual BRAT annotations. If a sentence contained an annotation or part of an annotation, that sentence was marked as true for that annotation, and false otherwise. A database of sentences was created, containing all the information and text about the sentence and the annotations for that sentence. The database was then split 80%/10%/10% into training, validation, and testing datasets. In order to prevent leakage of patient data between the sets, all of the sentences for an individual patient were placed into the same dataset. The split sizes were determined based on having as much training data as possible, given that some of the concepts were rare (highly unbalanced data). At the same time, the validation and testing sets needed to be large enough to perform a meaningful evaluation. Therefore, we made the training set as large as possible, while not making the other sets any smaller than 10% of the data. Counts of sentences and annotations assigned in each dataset are shown in Table 1.

**Table 1.** Annotation counts for the training, validation, and testing datasets.

	Training			Validation			Testing		
Concept	Positive	Negative qualified	Negative	Positive	Negative qualified	Negative	Positive	Negative qualified	Negative
Autonomic_dysfunction	69	35	88 343	31	11	30 811	32	9	28 519
Cerebral_palsy	153	46	88 248	132	36	30 685	83	31	28 446
Developmental_delay	739	593	87 115	279	182	30 392	245	163	28 152
Epilepsy_or_seizures	2881	2764	82 802	984	926	28 943	915	883	26 762
Feeding_issues	340	61	88 046	167	19	30 667	186	30	28 344
Hypotonia	201	636	87 610	91	187	30 575	86	170	28 304
Insomnia	122	80	88 245	23	4	30 826	32	10	28 518
Mood_disturbance	446	218	87 783	115	62	30 676	107	50	28 403
Movement_disorders	77	349	88 021	15	82	30 756	30	78	28 452
Oculogyric_crisis	45	10	88 392	8	4	30 841	7	0	28 553

#### Machine learning approach

Initial experimentation with the training dataset and a linear SVM classifier used unigram and bigram features from the dataset. It was found that there were not enough samples of negated, not patient, and hypothetical annotations to predict these categories separately. Therefore, these were combined into one single “negative qualified” category for each concept. The prediction task for each concept in each sentence was then defined as a 3-class prediction, consisting of the following 3 classes:

1. Negative—the concept is not present in the sentence
2. Negative qualified—the concept is present, but is either negated, not patient, and/or hypothetical.
3. Positive—the concept is present, and is not qualified as any of negated, not patient, or hypothetical.

All sentence-level classification tasks were then formulated as this 3-class problem.

### Machine learning concept algorithm optimization

In order to predict the 3 class AADCd concepts most accurately, a variety of alternative ML strategies were evaluated and compared using 5 repetitions of 2-way cross-validation on the training dataset.

Three types of features were evaluated: *n-*gram-based features, embedding vectors based on the pre-trained ClinicalBert model provided by HuggingFace (available at https://huggingface.co/emilyalsentzer/Bio_ClinicalBERT),^37^ and autoencoder-based features using various layer widths in a 5-stage encoder-decoder architecture. The *n*-gram features were obtained by parsing the sentences into *n*-grams of length one, two, or three tokens using the same spacy parser model used to divide the dataset into sentences. *N*-grams were then filtered for overall document frequency, and *n*-grams occurring in more than an upper threshold of the documents or less than a lower threshold of the documents were removed. The autoencoder experiments also used *n*-grams as input to a denoising autoencoder, which has been successful in prior reported biomedical text classification work.^38^^,^^39^

Cross-validation experiments using the support vector machine and logistic regression classifiers found that the best set of thresholds removed tokens that occurred in 95% of the documents or more, or less than 5% of training documents. Combining uni- and bi-grams resulted in improved performance, no performance gain was obtained by adding tri-grams. This resulted in 3952 *n-*gram-based token features.

Feature embedding vectors based on ClinicalBert were also evaluated. This model creates a feature vector from the entire sentence consisting of 768 dimensions. The *n*-gram and ClinicalBert feature vectors were then evaluated using cross-validation on the training set separately and concatenated into single vectors resulting in feature vectors of length 3952, 768, and 4720 respectively.

**Figure 2.**
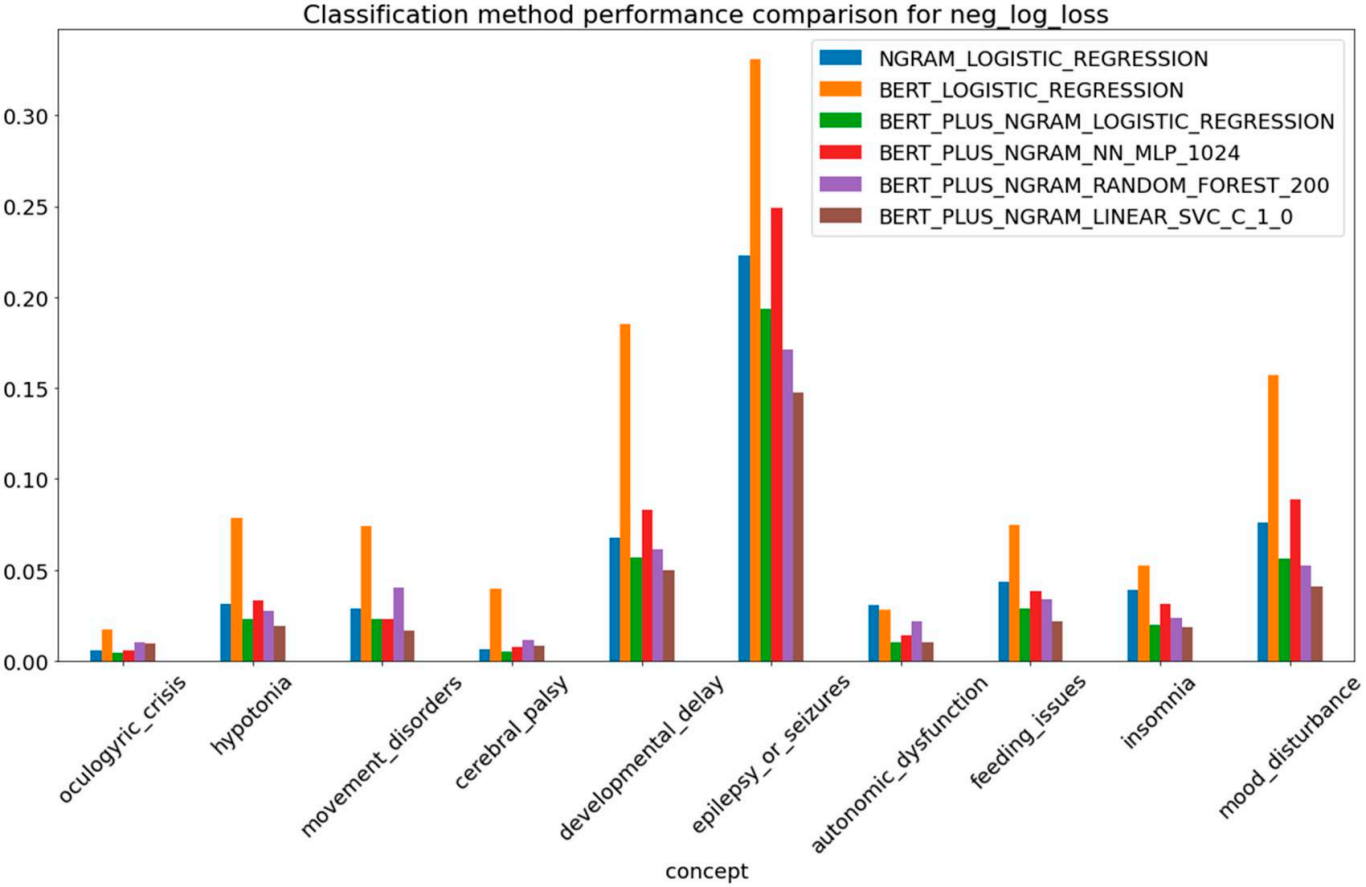
Negative log loss (smaller values are better) ML performance of representative examples of combinations of classifiers and feature sets evaluated by applying cross-validation on the training set. Abbreviations: NGRAM = uni- and bigram sentence features, BERT = ClinicalBert 768 dimension embedding vector, LOGISTIC_REGRESSION = logistic regression classifier in scikit-learn, default parameters, NN_MLP_1024 = neural network MLPClassifier in scikit-learn with hidden layer of size 1024, RANDOM_FOREST_200 = RandomForest classifier in scikit-learn with 200 estimators, other parameters at defaults, LINEAR_SVC_C_1.0 = support vector machine classifier, scikit-lean SVC implementation with C parameter set to 1.0.

Cross-validation on the training set was again applied using log-loss as the metric of accuracy. It was found that the combination of *n*-gram and ClinicalBert features consistently outperformed either feature type separately. The combined feature vector of 4720 dimensions consistently performed better than all other feature combinations as evaluated by cross-validation. It was determined that the SVM classifier performed as well as, or in most cases, better than the other classification approaches. The autoencoder-based features did not improve performance over the combination of *n*-gram and ClinicalBert features and as an individual feature set performed worse than *n*-grams. See Figure 2 for an example of comparisons that were evaluated.

The combined feature vectors were then used with several different classifier algorithms including SVM with multiple kernel types, random forest, logistic regression, 2 and 3-layer neural networks, and gradient boosting. The SVM-based kernels at default settings performed as well or better under cross-validation as the other algorithms, so SVM was chosen as the main algorithm, and kernel and parameter settings were optimized.

SVM with the linear kernel and the combined 4720 length feature vector was then used as a base for comparison with other SVM kernels. Kernel parameters were then optimized using grid search with combinations of the kernel, and parameter settings with the lowest log-loss were chosen for each AADCd concept. Negative down-sampling was also used to increase the concentration of positive samples in the training set for some concepts, with a downsampling range of 0.05 to 1.0 evaluated in steps of 0.05. See Table 2 for a list of the final ML models, kernels, and parameters chosen. The same vector of 4720 length consisting of the concatenated *n*-gram and ClinicalBert embedding features was used for all classifiers.

**Table 2.** Final optimized classifier parameters, down-sampling rate, and reduction method.

Concept	Kernel	Parameter	Reduction	Downsample	Sentence Validation + testing	
					AUC	AP
Autonomic_dysfunction	rbf	0.01	max	0.05	0.68	0.01
Cerebral_palsy	Linear	0.1	noisy_or	1.0	0.93	0.62
Developmental_delay	Linear	0.1	noisy_or	0.95	0.96	0.59
Epilepsy_or_seizures	Linear	0.01	noisy_or	1.0	0.94	0.57
Feeding_issues	Linear	0.01	noisy_or	0.90	0.93	0.33
Hypotonia	Linear	0.01	noisy_or	0.60	0.94	0.36
Insomnia	rbf	0.0001	noisy_or	0.60	0.90	0.02
Mood_disturbance	Linear	0.005	noisy_or	1.0	0.95	0.23
Movement_disorders	Linear	0.1	max	0.75	0.74	0.002
Oculogyric_crisis	Linear	8.0	max	1.00	0.93	0.002

Sentence level performance on the validation + testing datasets shown for AUC (area under the receiver operating curve) and AP (average precision).

Final predictive concept models were then created using these settings and the full training data set, split into 2/3 + 1/3 portions for model training and calibration with isotonic regression. The result of this step is a separate sentence-level predictive model for each concept giving the predictive probabilities for each of the 3 classes.

Performance was then evaluated on the validation dataset and checked for consistency with that predicted by the training dataset cross-validation selection procedure. The evaluated performance on the validation dataset was found to be consistent and close to the predicted performance. No changes were made to the models after the evaluation of the validation set. The final performance was then evaluated on the combined validation + testing dataset. See Table 2 for the performance of the final trained models on the validation + testing dataset.

### Combining concept sentence predictions into patient predictions

Patient-level training and validation datasets were then created for each concept by collapsing the annotated sentences for each patient into a single binary present/absent variable. If a patient had any positive manual annotation for a concept, that patient was assigned positive for that concept, otherwise assigned as negative. In this manner, a patient-concept-level gold standard was programmatically created from the individual sentence annotations.

Several methods were investigated to automatically combine the individual sentence-level predictions for a patient for a given concept into a single patient-level concept prediction. These methods were termed “reduction” functions, since they act like a reduction operation in functional programming, taking in a list of inputs (in this case sentence-level concept predicted probabilities) and outputting a single overall result (in this case, the patient-level concept predicted probability). The reduction functions evaluated included:

- max of positive sentence-level prediction probabilities
- min of positive sentence-level prediction probabilities
- mean of positive sentence-level prediction probabilities
- noisy-or of positive sentence-level prediction probabilities^40^
- two level neural networks trained on positive sentence-level prediction probabilities
- two level neural networks trained on positive, negative, and negative-qualified sentence-level prediction probabilities
- Linear SVM trained on positive sentence-level prediction probabilities
- Linear SVM trained on positive, negative, and negative-qualified sentence-level prediction probabilities

These methods were evaluated by comparing the algorithmic predictions with the gold standard. The best method for each concept was then chosen based on the performance on average precision. Average precision was chosen here as the best measure since the overall goal of the project is to rank patients for diagnostic screening for AADCd, and therefore it is reasonable to optimize the patient level predictions by the ability of the reduction function to rank patients for presence of the concepts of interest in their clinical notes. The best reduction algorithm and the patient concept performance obtained on the validation + testing data after choosing these settings on the training data set are shown in Table 2.

### Ranking patients by combining patient-level concept predictions

Finally, patient-level concept predictions were combined into patient-specific rank values for prioritizing manual review for AADCd screening. Since there is no “gold standard” for ranking patients in this manner, especially since the disease is very rare and there was a lack of appropriate cases to train on, this was done in 2 steps. First, it was postulated that patients having a higher number of AADCd-related symptoms would be more likely to be good screening candidates. Therefore, an overall target rank score was calculated for each patient in the training data based simply on the count of the number of positive symptoms that they had in their gold standard patient-level concept set.

This overall target rank count included all concepts except for *Epilepsy or Seizures*, which has a complex relationship with AADCd and was handled differently from the other concepts. *Epilepsy or Seizures*, while a distinct condition in itself, can be related to AADCd in 3 ways: (1) patients with AADCd have isolated seizures as part of the clinical presentation (seizures occur more frequently in AADCd than the general population), (2) oculogyric crises can be misdiagnosed as seizures, and (3) patients with AADCd can have both oculogyric crises And seizures as part of the clinical presentation.

It has been estimated that 4.5% to 8% of AADCd patients also have seizures/epilepsy,^3^^,^^10^ which is more common than in the general population. While 68% of AADCd patients experience oculogyric crisis, this can be confused for seizures.^41^

In the second step, a Poisson regression model was created that took as input in the patient-level concept probabilities (all of them, including epilepsy as a predictive variable) and was fit to predict the number of counted AADCd symptoms assigned in the first step. This predicted symptom count was then used as the patient ranking value for manual review.

**Figure 3.**
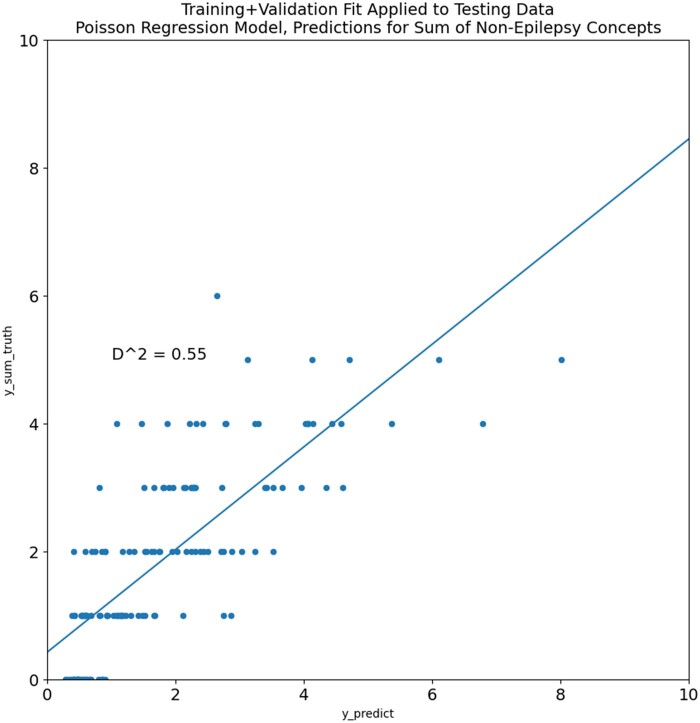
Poisson regression model combining all individual concept predictors and fit to the actual count of the concept occurrences for each patient in the training + validation set. Plotted points are the predicted and actual occurrence counts for the testing dataset.

Initially, with this method, the Poisson regression was performed on the training data, and the fit was compared to the validation dataset. After this step demonstrated a good fit with a *D*-squared value of 0.63, the Poisson regression was fit on the training + validation data, and this is the final regression ranking model used in our approach on unseen data. The mean symptom count on the training + validation data was 1.615, with a standard deviation of 1.472. Performance of the model fit on the training + validation data and tested on the test data is shown in Figure 3. The final coefficients of the model fit on the training + validation data were as follows:

- Autonomic_dysfunction coefficient = 0.008
- Cerebral_palsy coefficient = 0.432
- Developmental_delay coefficient = 1.299
- Epilepsy_or_seizures coefficient = −0.426
- Feeding_issues coefficient = 0.705
- Hypotonia coefficient = 0.351
- Insomnia coefficient = 0.283
- Mood_disturbance coefficient = 0.565
- Movement_disorders coefficient = 0.508
- Oculogyric_crisis coefficient = 0.919

The methodology above results in a set of models and applicable steps that can be applied to unseen data, and that produce ranking values for each individual patient.

### Evaluation approach

We applied the methodology described in the previous sections to the 8025 patients in the held-out partitions 1-9. These patients comprised all unseen data that neither the investigators nor the algorithms had seen before. The top-ranked and bottom-ranked 200 patients were then identified, randomly ordered, and their clinical notes were manually reviewed. The review was done blinded by the annotation team, while annotating they had no information about the overall rank score or the individual concept predictions for any patient. Each patient was noted as to whether patients had AADCd compatible symptoms, whether these symptoms already had a definitive diagnosis expressed in the chart, and finally, if the first was true and the second false, whether the patient was an appropriate candidate for the next phase of AADCd screening. This last criterion was used as the outcome variable for the evaluation. For this work, a manual chart review of algorithm-identified patients by the epidemiologist annotator was taken as the endpoint of the study.

Note that this manual screening of the high- and low-ranked patients sets a higher-utility criterion for the results obtained by the algorithm. The ML approach was not specifically trained to recognize non-AADCd diseases or conditions that could explain the symptoms. It was considered important to base this final evaluation on the end goal of the project—identifying potentially undiagnosed cases of AADCd, and therefore setting an evaluation criterion that includes consideration of the overall purpose would allow us to evaluate a lower bar of the true performance of the approach.

## Results

### Concept-level scoring on patients

The performance of the individual concept recognizers, combined with the reduction functions, evaluated on the individual patients in the validation set are shown in Table 3. The average precision obtained ranged from 0.11 for insomnia to 0.99 for epilepsy. The lift obtained (average precision divided by prevalence) was above 1.0 for all concepts, and often higher, demonstrating that there is some discriminative value for all concept classifiers, even the ones applied to relatively rare concepts such as oculogyric crisis and movement disorders. While sentence-level prediction performance can be low for some concepts, especially the rarest, the reduction process elevated the patient-level predictions to a more useful level of accuracy.

**Table 3.** Results of applying concept level classification and patient reduction functions to the individual AADC related concepts on the validation + testing dataset showing average-precision and lift for each concept.

	Validation + testing data				
Concept	n_subjects	n_positive	prevalence	AP	LIFT
Autonomic_dysfunction	291	34	0.116838	0.197217	1.687948
Cerebral_palsy	291	67	0.230241	0.948605	4.120062
Developmental_delay	291	157	0.539519	0.941920	1.745852
Epilepsy_or_seizures	291	205	0.704467	0.988592	1.403318
Feeding_issues	291	105	0.360825	0.837520	2.321126
Hypotonia	291	54	0.185567	0.680023	3.664567
Insomnia	291	24	0.082474	0.109655	1.329566
Mood_disturbance	291	93	0.319588	0.743259	2.325683
Movement_disorders	291	12	0.041237	0.144334	3.500097
Oculogyric_crisis	291	13	0.044674	0.112893	2.527056

N_subjects are patients in the validation dataset, n_positive are the number of patients with a positive sentence for that concept. Prevalence is n_positive/n_subjects. Abbreviations: AP = average precision, LIFT = average_precision/prevalence.

### Patient-level scoring and ranking

**Figure 4.**
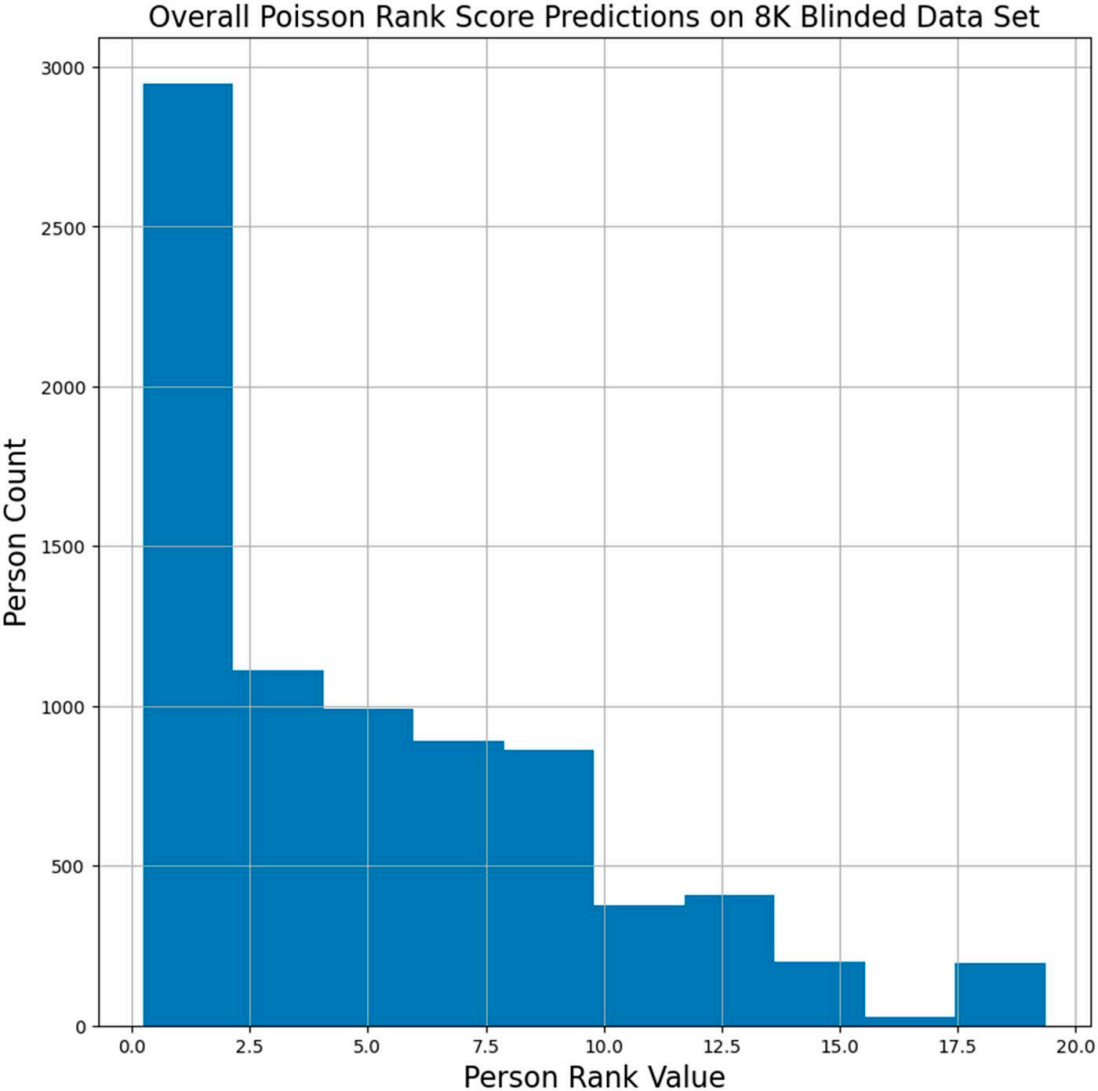
Predicted overall Poisson rank score distribution of the final algorithm for all 8010 patients in the blinded dataset. The mean predicted rank value was 5.136 with a minimum score of 0.246 and a maximum score of 19.351. The 25%/50%/75% percentile boundaries were at 1.172, 3.962, and 8.066, respectively. The top 200 patients had rank scores of 17.260 or higher.

The results of applying the concept scoring and Poisson regression rank value calculation to the 8010 patients in our test group are shown as a histogram in Figure 4. The mean predicted rank value was 5.136 with a minimum score of 0.246 and a maximum score of 19.351. The 25%/50%/75% percentile boundaries were at 1.172, 3.962, and 8.066, respectively. The top 200 patients had rank scores of 17.260 or higher.

**Figure 5.**
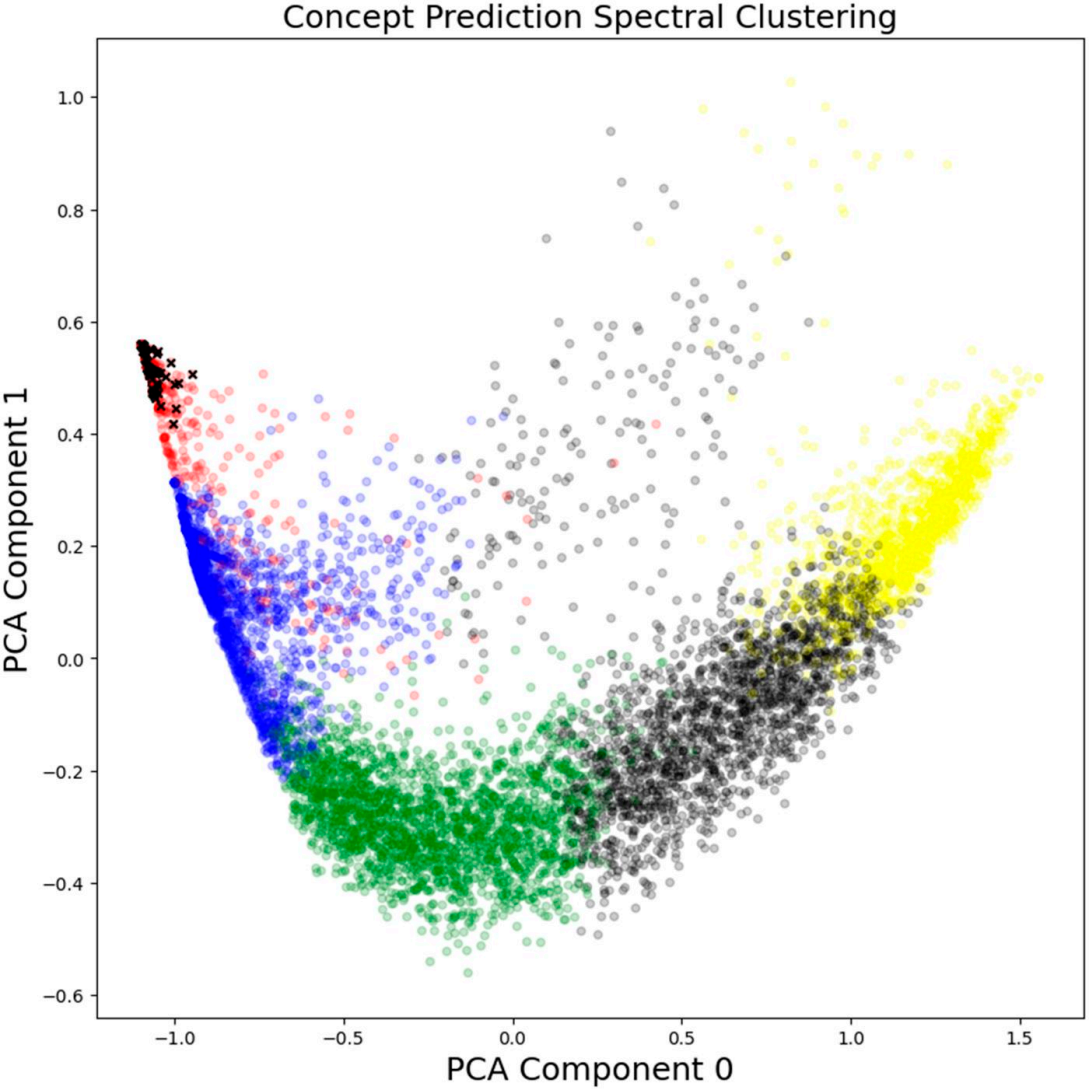
Spectral clustering analysis of ML concept predictions and correspondence with top 200 ranked patients in the blinded dataset. Separate clusters are shown as groups of similarly shaded and colored circles and plotted in 2 dimensions based on principle component analysis of the 10 concept prediction values for each patient. Patients ranked in the top 200 by the overall Poisson score are also shown as crosses. All the top-ranked patients fall in the upper left corner of the upper left-most (red) cluster.

To investigate the ability of our concept classifiers to separate patients into meaningful categories, and in order to study the clinical profile of the patients ranked in the top 200, spectral clustering was performed on the 8010 patient concept scores, using the “SpectralClustering” package in scikit-learn.^42^ Visual inspection examining clustering with 3 through 8 groups, showed that 5 clusters gave the best group separation for the smallest number of clusters which had approximately the same number of patients and had no tiny clusters. Two-dimensional principal component analysis (PCA) of the individual concept scores is shown in Figure 5 with the clusters plotted as separate colors. Patients in the top 200 rank scores are plotted as x’s in black. All other samples are plotted as circles in black, blue, red, green, or yellow. It is clear from the figure that all the top 200 ranked patients fall into the red cluster, which represents a low score in component 0 and a high score in component 1. This is an interesting validation of the proposed ranking method since the spectral clustering and PCA analysis did not include the Poisson regression rank value as a clustering feature, only the individual predicted concept probabilities

### Manual case review evaluation

As described above, 400 patient records were manually reviewed and assessed for appropriateness for the next phase of diagnostic screening. The results of the assessment are shown in Table 4. A total of 45 of the 200 top-ranked cases were characterized as requiring clinical screening for manual review, this is a positive predictive value of 22.5%. None of the bottom-ranked patients were characterized as appropriate for diagnostic screening. This difference is statistically significant by both Fisher’s exact and chi-square test with Yeats correction at *P* ≪ .0001.

**Table 4.** Manual review of the top 200 and bottom 200 patients ranked by the automated approach.

	Requires clinician review?	
Group	No	Yes
Bottom 200	200	0
Top 200	155	45

Patients in the top 200 are much more likely to pass this screening and be forwarded for clinician review than those in the bottom 200. Fisher’s exact: *P* ≪ .0001, chi-square with Yeats: *P* ≪ .0001.

## Discussion

Our results showed that the proposed method worked well to identify patients in this population where diagnostic testing for AADCd is potentially indicated. Almost 23% of the top-ranked cases were marked for review by a clinician disease expert and for potential diagnostic testing. None of the lowest-ranked patients were designated for clinician review. This result was statistically significant to a high degree.

Our algorithm could be enhanced with structured data as a way to filter out patients who already have an explanatory diagnosis for the symptoms of interest. It is possible to define a set of diagnostic codes that could be used to filter out patients who already had sufficient reasons for their symptoms. This would take expert judgment to decide which conditions were sufficiently explanatory on their own, and which could cooccur with a disease such as AADCd.

The method presented here is based on the OMOP data model; in particular, we made heavy use of the OMOP NOTE table as well as the PERSON table for demographic cohort selection. Therefore, this approach and implementation are not dependent upon any particular EHR system, and it should be reasonably straightforward to move our methods to other sites.

Our approach could be expanded and enhanced to work for other rare and semi-rare diseases since it is focused on automatically identifying sets of relatively common symptoms and does not require training data consisting of records of patients with known rare diseases. The method should generalize to other conditions where a set of related symptoms and conditions can be created, is usually mentioned in the note and other text of the medical record and not in the coded data, and cases are rare in the EHR and unavailable for direct training on diagnosed cases. This could also be a valuable approach for uncommon, not necessarily rare, diseases that have alternate or atypical presentations. For example, celiac disease, while commonly presenting with GI symptoms based on an immunologic response to gluten, also can present with non-GI symptoms in addition to, or without GI symptoms. These atypical symptoms include headache, peripheral neuropathy, and cerebellar ataxia, may be present in the chart notes, and are unlikely to be coded as structured data.^43^^,^^44^

Furthermore, the approach could be expanded to address sets of rare diseases and not one rare disease at a time. A larger set of symptoms and related conditions could cover a range of diseases, such as many inborn errors of neurotransmitter metabolism and not just AADCd. Future work will investigate what set of symptoms and conditions would provide good coverage for an expanded set of diseases, and how to best determine which symptoms and conditions would be most useful and reusable across a range of diseases.

Our methods used traditional supervised learning NLP/ML. One question is whether large language models (LLMs) such as GPT4 may have an impact on biomedical text research and future clinical NLP applications.^45^ Both large commercial models (such as GPT4) as well as LLMs small enough to be run locally, such as Llama,^46^ Alpaca,^47^ the huggingface (https://huggingface.co/), and GPT4All (https://gpt4all.io/index.html) collections of models have recently become widely available. Running commercial models on clinical text may have some initial barriers, such as requiring a business partner agreement to ensure privacy, security protection, and Health Insurance Portability and Accountability Act (HIPAA) compliance, which would take some time to set up and govern. There are also issues with a lack of user control of changes in commercial models (eg, tuning on one version, with performance changing on the next, with no access to the prior version of the model).

The smaller, localizable LLMs can be run completely inside a healthcare institutional firewall, decreasing the HIPAA concerns. Given the large difference in available resources, it is unlikely that the localizable models will be able to keep up in performance with the ever-growing parameter size and training base of the commercial models.^48^ However, local models can be tuned for individual institutional requirements. This can be a substantial, but still currently tractable task for healthcare research institutions with the right available hardware.^49–51^

Performance between the LLMs varies with model, prompt, as well as task, sometimes in very unpredictable ways.^52–54^ Because local LLMs with good performance in clinical medicine have only recently become generally available (eg, GatorTron^55^) we haven’t applied these models yet in our AADCd research, but plan to in the future. In theory, the LLMs could produce probabilistic estimates of patient symptoms on spans of clinical text. Some researchers do not think that LLMs can provide accurate confidence estimates of their output.^56^ Breaking down a complex diagnosis into individual symptoms, as has been done here, is likely to produce better near-term results as compared to asking a complex synthesis question such as “Should the patient be screened for AADCd?”.^53^ While these LLMs do not require annotated training data in the traditional sense, they do require detailed, and sometimes brittle^57^ prompt engineering to coerce the models to produce the correct output. Annotated data for evaluation is also necessary to guide prompt engineering and also to do evaluation in order to determine how well they perform in comparison to traditionally trained methods. Prompt engineering in particular is a new emerging field, and it will take some time before the best methods of creating prompts for specific tasks are established.^57^ Since the LLMs have been shown to have problems with “hallucination” and “confabulation,”^58^^,^^59^ evaluation of extraction probabilistic confidence accuracy is also needed.

The work presented here has several limitations. First, all the data were sourced from a single health system, and the text in the notes may reflect documentation practices from that site. It is likely that the concept classifiers built here would decrease in performance on data from other sites and may improve with some site-specific training data. Going forward, it is important to replicate these findings on outside datasets. Currently, these are not available to us. The most widely publicly available clinical datasets, MIMIC II-IV,^60^^,^^61^ are based primarily on critical-care patients and not on the pediatric neurology population focused on here. Collaborating with another healthcare institution with an OMOP-compliant RDW would be one feasible way to achieve this goal.

Second, all the chart review was performed by a trained epidemiologist and not a rare disease expert. While review by clinical experts would be the next step, those resources were not available in this study. In future work, we intend to incorporate clinician expert manual chart review by a pediatric neurologist, and any systematic difference in manual review between the epidemiologist and neurologist will be analyzed. As none of the patients screened had a diagnosis of AADCd in their chart, determining an actual diagnosis of AADCd will require laboratory testing of patients after pediatric neurologist screening.

## Conclusion

The work presented here has demonstrated a novel, feasible, generalizable approach for detecting potential undiagnosed cases of rare diseases in large population EHR systems, applied to the specific rare disease of AADCd. Future work will enhance the approach to a wider range of diseases, include structured EHR data for patient filtering, and follow up the current research with a detailed clinician review of selected patients. It is also our goal to collaborate with other institutions to apply our methods to additional populations that have an OMOP-based RDW.

## Supplementary Material

ocad244_Supplementary_Data

## Data Availability

The source data used for this project is EHR data and contains protected health information (PHI) for patients under care at Oregon Health & Science University (OHSU). Trained models may also include PHI as features. The OHSU Institutional Review Board (IRB) does not allow the release of this data to the public, and doing so would violate US HIPAA laws. The OHSU IRB can be contacted at: irb@ohsu.edu. Questions about data requests may be sent to this address. We are including full details of the ML model, training methods, and hyperparameter settings. Other investigators experienced in the field should be able to reproduce our methods on their own data to validate the results presented in this manuscript.

## References

[1] Pearson TS , Gilbert L, Opladen T, et al AADC deficiency from infancy to adulthood: symptoms and developmental outcome in an international cohort of 63 patients. J Inherit Metab Dis. 2020;43(5):1121-1130. 32369189 10.1002/jimd.12247 PMC7540529

[2] Himmelreich N , Bertoldi M, Alfadhel M, et al Prevalence of DDC genotypes in patients with aromatic L-amino acid decarboxylase (AADC) deficiency and in silico prediction of structural protein changes. Mol Genet Metab. 2023;139(4):107647. 37453860 10.1016/j.ymgme.2023.107647

[3] Rizzi S , Spagnoli C, Frattini D, Pisani F, Fusco C. Clinical features in aromatic L-Amino acid decarboxylase (AADC) deficiency: a systematic review. *Behav Neurol*. 2022;2022:2210555. 10.1155/2022/2210555 PMC9578880 36268467

[4] Gowda VK , Vegda H, Nagarajan BB, Shivappa SK. Clinical profile and outcome of Indian children with aromatic L-amino acid decarboxylase deficiency: a primary CSF neurotransmitter disorder mimicking as dyskinetic cerebral palsy. J Pediatr Genet. 2021;10(2):85-91. 33996177 10.1055/s-0040-1714690 PMC8110350

[5] Helman G , Pappa MB, Pearl PL. Widening phenotypic spectrum of AADC deficiency, a disorder of dopamine and serotonin synthesis. JIMD Rep.2014;17:23-27. 25001633 10.1007/8904_2014_327 PMC4241195

[6] Moreno-De-Luca A , Millan F, Pesacreta DR, et al Molecular diagnostic yield of exome sequencing in patients with cerebral palsy. JAMA 2021;325(5):467-475. 33528536 10.1001/jama.2020.26148 PMC7856544

[7] Zouvelou V , Yubero D, Apostolakopoulou L, et al The genetic etiology in cerebral palsy mimics: the results from a Greek tertiary care center. Eur J Paediatr Neurol. 2019;23(3):427-437. 30799092 10.1016/j.ejpn.2019.02.001

[8] Lewis SA , Shetty S, Wilson BA, et al Insights from genetic studies of cerebral palsy. *Front Neurol*. 2020;11:625428. 10.3389/fneur.2020.625428 PMC7859255 33551980

[9] Manegold C , Hoffmann GF, Degen I, et al Aromatic L‐amino acid decarboxylase deficiency: clinical features, drug therapy and follow‐up. J Inherit Metab Dis. 2009;32(3):371-380. 19172410 10.1007/s10545-009-1076-1

[10] Wassenberg T , Molero-Luis M, Jeltsch K, et al Consensus guideline for the diagnosis and treatment of aromatic L-amino acid decarboxylase (AADC) deficiency. Orphanet J Rare Dis. 2017;12(1):12-21. 28100251 10.1186/s13023-016-0522-z PMC5241937

[11] Haendel M , Vasilevsky N, Unni D, et al How many rare diseases are there? Nat Rev Drug Discov. 2020;19(2):77-78. 32020066 10.1038/d41573-019-00180-y PMC7771654

[12] Schaefer J , Lehne M, Schepers J, Prasser F, Thun S. The use of machine learning in rare diseases: a scoping review. Orphanet J Rare Dis. 2020;15(1):145. 32517778 10.1186/s13023-020-01424-6 PMC7285453

[13] Visibelli A , Roncaglia B, Spiga O, Santucci A. The impact of artificial intelligence in the odyssey of rare diseases. Biomedicines 2023;11(3):887. 36979866 10.3390/biomedicines11030887 PMC10045927

[14] Elder G , Harper P, Badminton M, Sandberg S, Deybach JC. The incidence of inherited porphyrias in Europe. J Inherit Metab Dis. 2013;36(5):849-857. 23114748 10.1007/s10545-012-9544-4

[15] Bonkovsky HL , Maddukuri VC, Yazici C, et al Acute porphyrias in the USA: features of 108 subjects from porphyrias consortium. Am J Med. 2014;127(12):1233-1241. 25016127 10.1016/j.amjmed.2014.06.036 PMC4563803

[16] Cohen AM , Chamberlin S, Deloughery T, et al Detecting rare diseases in electronic health records using machine learning and knowledge engineering: case study of acute hepatic porphyria. PLoS One. 2020;15(7):e0235574. 32614911 10.1371/journal.pone.0235574 PMC7331997

[17] Hersh WR , Cohen AM, Nguyen MM, Bensching KL, Deloughery TG. Clinical study applying machine learning to detect a rare disease: results and lessons learned. JAMIA Open. 2022;5(2):ooac053. 35783073 10.1093/jamiaopen/ooac053 PMC9243401

[18] Garg R , Dong S, Shah S, Jonnalagadda SA. Bootstrap machine learning approach to identify rare disease patients from electronic health records. arXiv, arXiv:1609.01586. http://arxiv.org/abs/1609.01586, 2016, preprint: not peer reviewed.

[19] Jamian L , Wheless L, Crofford LJ, Barnado A. Rule-based and machine learning algorithms identify patients with systemic sclerosis accurately in the electronic health record. Arthritis Res Ther. 2019;21(1):305. 31888720 10.1186/s13075-019-2092-7 PMC6937803

[20] Colbaugh R , Glass K. Finding Rare Disease Patients in EHR Databases via Lightly-Supervised Learning. medRxiv 2020.07.06.20147322v1. https://www.medrxiv.org/content/10.1101/2020.07.06.20147322v1, 2020, preprint: not peer reviewed.

[21] Hully M , Lo Barco T, Kaminska A, et al Deep phenotyping unstructured data mining in an extensive pediatric database to unravel a common KCNA2 variant in neurodevelopmental syndromes. Genet Med. 2021;23(5):968-971. 33500571 10.1038/s41436-020-01039-z PMC8105164

[22] Dros JT , Bos I, Bennis FC, et al Detection of primary Sjögren’s syndrome in primary care: developing a classification model with the use of routine healthcare data and machine learning. BMC Prim Care. 2022;23(1):199. 35945489 10.1186/s12875-022-01804-w PMC9361661

[23] Lo Barco T , Kuchenbuch M, Garcelon N, Neuraz A, Nabbout R. Improving early diagnosis of rare diseases using natural language processing in unstructured medical records: an illustration from Dravet syndrome. Orphanet J Rare Dis. 2021;16(1):309. 34256808 10.1186/s13023-021-01936-9 PMC8278630

[24] Faviez C , Vincent M, Garcelon N, et al Enriching UMLS-based phenotyping of rare diseases using deep-learning: evaluation on Jeune syndrome. Stud Health Technol Inform. 2022;294:844-848. 35612223 10.3233/SHTI220604

[25] Barnado A , Eudy AM, Blaske A, et al Developing and validating methods to assemble systemic lupus erythematosus births in the electronic health record. Arthritis Care Res (Hoboken). 2022;74(5):849-857. 33253488 10.1002/acr.24522 PMC8164642

[26] Chen X , Faviez C, Vincent M, et al Patient-patient similarity-based screening of a clinical data warehouse to support ciliopathy diagnosis. Front Pharmacol. 2022;13:786710. 35401179 10.3389/fphar.2022.786710 PMC8993144

[27] Lin S , Nateqi J, Weingartner-Ortner R, et al An artificial intelligence-based approach for identifying rare disease patients using retrospective electronic health records applied for Pompe disease. Front Neurol. 2023;14:1108222. 37153672 10.3389/fneur.2023.1108222 PMC10160659

[28] Michalski AA , Lis K, Stankiewicz J, et al Supporting the diagnosis of Fabry disease using a natural language processing-based approach. J Clin Med. 2023;12(10):3599. 37240705 10.3390/jcm12103599 PMC10219252

[29] Hwu WL , Chien YH, Lee NC, Li MH. Natural history of aromatic L-amino acid decarboxylase deficiency in Taiwan. JIMD Rep. 2018;40:1-6. 28856607 10.1007/8904_2017_54 PMC6122029

[30] Pons R , Ford B, Chiriboga CA, et al Aromatic L-amino acid decarboxylase deficiency: clinical features, treatment, and prognosis. Neurology. 2004;62(7):1058-1065. 15079002 10.1212/wnl.62.7.1058

[31] Helman G , Pappa MB, Pearl PL. Erratum to: widening phenotypic spectrum of AADC deficiency, a disorder of dopamine and serotonin synthesis. JIMD Rep. 2014;17:97. 25944030 10.1007/978-3-662-44578-5_344 PMC6373175

[32] Dai W , Lu D, Gu X, Yu Y, Disease MCL of AR Aromatic L‐amino acid decarboxylase deficiency in 17 mainland China patients: clinical phenotype, molecular spectrum, and therapy overview. Mol Genet Genomic Med. 2020;8(3):e1143. 31975548 10.1002/mgg3.1143 PMC7057092

[33] Reinecke I , Zoch M, Reich C, Sedlmayr M, Bathelt F. The usage of OHDSI OMOP - a scoping review. *Stud Health Technol Inform*. 2021;283:95-103. 10.3233/SHTI210546 34545824

[34] Klann JG , Phillips LC, Herrick C, Joss MAH, Wagholikar KB, Murphy SN. Web services for data warehouses: OMOP and PCORnet on i2b2. J Am Med Inform Assoc. 2018;25(10):1331-1338. 30085008 10.1093/jamia/ocy093 PMC6188504

[35] Klann JG , Joss MA, Embree K, Murphy SN. Data model harmonization for the all of us research program: transforming i2b2 data into the OMOP common data model. PLoS One. 2019;14(2):e0212463. 30779778 10.1371/journal.pone.0212463 PMC6380544

[36] Stenetorp P , Pyysalo S, Topić G, Ohta T, Ananiadou S, Tsujii J. BRAT: a web-based tool for NLP-assisted text annotation. In: *Proceedings of the Demonstrations at the 13th Conference of the European Chapter of the Association for Computational Linguistics*. Avignon, France: Association for Computational Linguistics; 2012:102-107.

[37] Alsentzer E , Murphy JR, Boag W, et al Publicly Available Clinical BERT Embeddings. In: *Proceedings of the 2nd Clinical Natural Language Processing Workshop*. Minneapolis, MN: Association for Computational Linguistics; 2019:72-78.

[38] Vincent P , Larochelle H, Lajoie I, Bengio Y, Manzagol PA, Bottou L. Stacked denoising autoencoders: Learning useful representations in a deep network with a local denoising criterion. J Mach Learn Res. 2010;11(12).

[39] Kontonatsios G , Spencer S, Matthew P, Korkontzelos I. Using a neural network-based feature extraction method to facilitate citation screening for systematic reviews. Exp Syst Applicat: X. 2020;6:100030.

[40] Cozman FG. Axiomatizing noisy-OR. In: *Proceedings of the 16th European Conference on Artificial Intelligence*, Valencia, Spain. August 22, 2004:981-982.

[41] Ito S , Nakayama T, Ide S, et al Aromatic L‐amino acid decarboxylase deficiency associated with epilepsy mimicking non‐epileptic involuntary movements. Dev Med Child Neurol. 2008;50(11):876-878. 18754761 10.1111/j.1469-8749.2008.03094.x

[42] Von Luxburg U. A tutorial on spectral clustering. Stat Comput. 2007;17(4):395-416.

[43] Celiloğlu C , Karabiber H, Selimoğlu MA. Atypical presentations of celiac disease. Turk J Pediatr. 2011;53(3):241-249. 21980803

[44] Admou B , Essaadouni L, Krati K, et al Atypical celiac disease: from recognizing to managing. Gastroenterol Res Pract. 2012;2012:637187. doi:10.1155/2012/637187. 22811701 PMC3395124

[45] Arora A , Arora A. The promise of large language models in health care. Lancet. 2023;401(10377):641. 10.1016/S0140-6736(23)00216-7 36841609

[46] Touvron H , Lavril T, Izacard G, et al LLaMA: open and efficient foundation language models. arXiv, arXiv:2302.13971. http://arxiv.org/abs/2302.13971, 2023, preprint: not peer reviewed.

[47] Taori R , Gulrajani I, Zhang T, et al Alpaca: a strong, replicable instruction-following model. Stanf Center Res Found Mod. 2023;3(6):7. https://crfmstanfordedu/2023/03/13/alpacahtml

[48] Kaplan J , McCandlish S, Henighan T, et al Scaling laws for neural language models. arXiv, arXiv:2001.08361. http://arxiv.org/abs/2001.08361, 2020, preprint: not peer reviewed.

[49] Li C , Wong C, Zhang S, et al LLaVA-Med: training a large language-and-vision assistant for biomedicine in one day. arXiv, arXiv:2306.00890. http://arxiv.org/abs/2306.00890, 2023, preprint: not peer reviewed.

[50] Xiong H , Wang S, Zhu Y, et al DoctorGLM: fine-tuning your Chinese doctor is not a Herculean task. arXiv, arXiv:2304.01097. http://arxiv.org/abs/2304.01097, 2023, preprint: not peer reviewed.

[51] Hu EJ , Shen Y, Wallis P, et al LoRA: low-rank adaptation of large language models. arXiv, arXiv:2106.09685. http://arxiv.org/abs/2106.09685, 2021, preprint: not peer reviewed.

[52] Zhao Z , Wallace E, Feng S, Klein D, Singh S. Calibrate before use: improving few-shot performance of language models. In: *International Conference on Machine Learning 2021*. PMLR; July 1, 2021:12697-12706. Virtual Only Conference.

[53] Liu P , Yuan W, Fu J, Jiang Z, Hayashi H, Neubig G. Pre-train, prompt, and predict: a systematic survey of prompting methods in natural language processing. ACM Comput Surv. 2023;55(9):1-35.

[54] Bommasani R , Liang P, Lee T. Holistic evaluation of language models. *Ann N Y Acad Sci*. 2023;1525(1):140-146. 10.1111/nyas.15007 37230490

[55] Yang X , Chen A, PourNejatian N, et al A large language model for electronic health records. NPJ Digit Med. 2022;5(1):194. 36572766 10.1038/s41746-022-00742-2 PMC9792464

[56] Bender EM , Koller A. Climbing towards NLU: on meaning, form, and understanding in the age of data. In: *Proceedings of the 58th Annual Meeting of the Association for Computational Linguistics*. Association for Computational Linguistics. 2020:5185–5198. Accessed August 14, 2023. https://aclanthology.org/2020.acl-main.463

[57] Lu Y , Bartolo M, Moore A, Riedel S, Stenetorp P. Fantastically ordered prompts and where to find them: Overcoming few-shot prompt order sensitivity. arXiv, arXiv:210408786. 10.1101/210408786. 2021, preprint: not peer reviewed.

[58] Ali R , Tang OY, Connolly ID, et al Performance of ChatGPT, GPT-4, and Google bard on a neurosurgery oral boards preparation question bank. *Neurosurgery*. 2023;93(5):1090-1098. 10.1227/neu.0000000000002551 37306460

[59] Ang TL , Choolani M, See KC, Poh KK. The rise of artificial intelligence: addressing the impact of large language models such as ChatGPT on scientific publications. Singapore Med J. 2023;64(4):219-221. 37006087 10.4103/singaporemedj.SMJ-2023-055 PMC10144457

[60] Johnson AEW , Bulgarelli L, Shen L, et al MIMIC-IV, a freely accessible electronic health record dataset. Sci Data. 2023;10(1):31. 36596836 10.1038/s41597-022-01899-x PMC9810617

[61] Goldberger AL , Amaral LA, Glass L, et al PhysioBank, PhysioToolkit, and PhysioNet: components of a new research resource for complex physiologic signals. Circulation. 2000;101(23):e215-e220. 10851218 10.1161/01.cir.101.23.e215

