# Supplementary material for: Automatically pre-screening patients for the rare disease aromatic l-amino acid decarboxylase deficiency using knowledge engineering, natural language processing, and machine learning on a large EHR population
Source: J Am Med Inform Assoc. 2023 Dec 22;31(3):692–704. doi: 10.1093/jamia/ocad244 (PMC10873832; doi:10.1093/jamia/ocad244)
Supplement: ocad244_Supplementary_Data [file ocad244_supplementary_data.docx]

Supplemental Appendix

**For *Automatically Pre-Screening Patients for the Rare Disease Aromatic L-amino Acid Decarboxylase (AADC) Deficiency Using Knowledge Engineering, Natural Language Processing and Machine Learning on a Large EHR Population.***

**
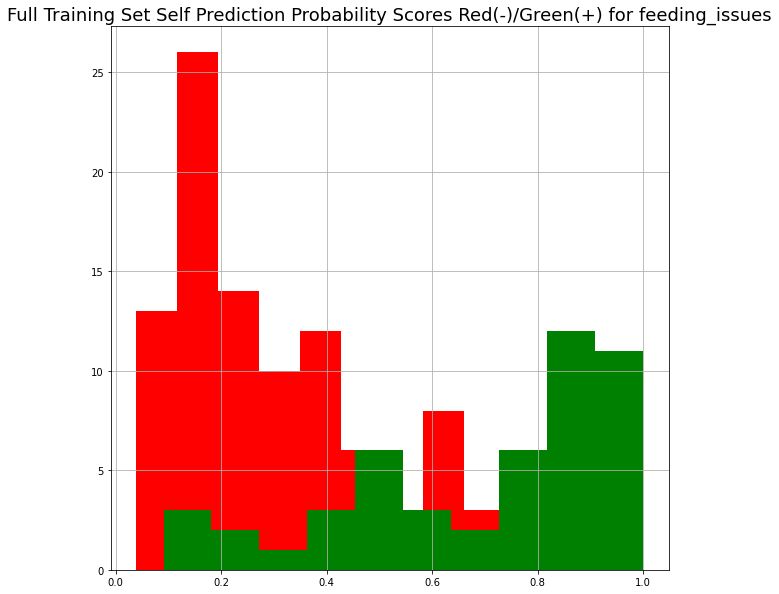

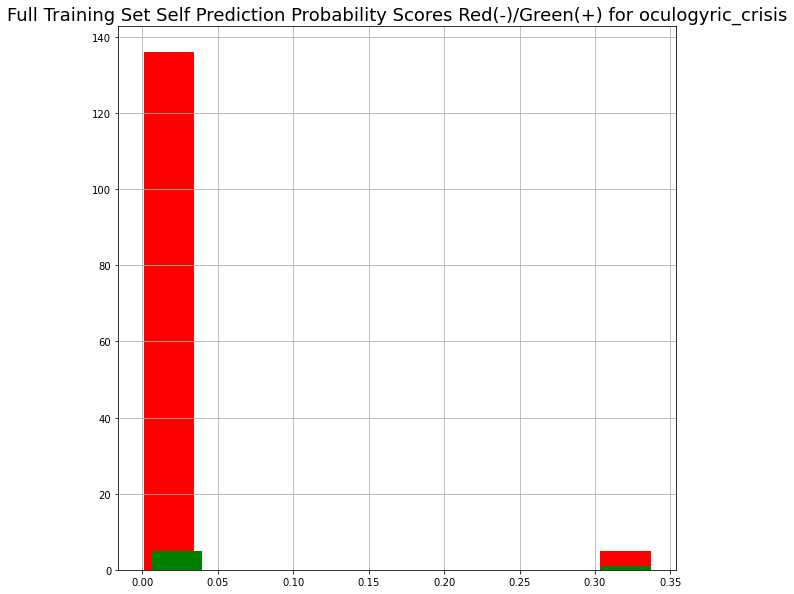
**

**Figures and tables not referenced in the main manuscript.**

**Table S1.** Cluster mean scores for each component concept. The red cluster contains all our highest ranked subjects. The red cluster also had the highest overall Poisson ranking score mean value. Overall subject Poisson ranking score was NOT used in the clustering process.

| **concept** | **RED** | **GREEN** | **BLUE** | **YELLOW** | **BLACK** |
| --- | --- | --- | --- | --- | --- |
| **autonomic_dysfunction** | **0.002773** | **0.002640** | **0.003020** | **0.002538** | **0.003464** |
| **cerebral_palsy** | **0.283777** | **0.164623** | **0.885105** | **0.051772** | **0.909921** |
| **developmental_delay** | **0.938524** | **0.651670** | **0.995456** | **0.218307** | **0.995282** |
| **epilepsy_or_seizures** | **0.985721** | **0.938249** | **0.998886** | **0.638563** | **0.998590** |
| **feeding_issues** | **0.880925** | **0.455554** | **0.980170** | **0.181919** | **0.991513** |
| **hypotonia** | **0.738277** | **0.272303** | **0.961172** | **0.127273** | **0.979433** |
| **insomnia** | **0.560451** | **0.192803** | **0.845352** | **0.085634** | **0.909643** |
| **mood_disturbance** | **0.930525** | **0.585677** | **0.982892** | **0.284513** | **0.993607** |
| **movement_disorders** | **0.113478** | **0.055470** | **0.205450** | **0.031629** | **0.887228** |
| **oculogyric_crisis** | **0.039746** | **0.026049** | **0.101440** | **0.015025** | **0.141817** |
| **overall_poisson** | **14.523580** | **4.837060** | **9.371358** | **0.562072** | **1.540494** |
| **count** | **2245** | **2020** | **1895** | **1250** | **600** |

**Figure S1.** Group by group plot of scores vs. character count for top 200 ranked patients. There is little correlation between the total character count of a patient’s notes and the overall score of the proposed ranking algorithm. There is also no separation between patients designated by the epidemiologist as requiring clinician review (red dots) and those not so designated (black dots) based on character count. Using a character count threshold would have missed many of the patients designated as requiring clinician review. This demonstrates that the proposed symptom-based algorithm discriminates much better than character count for designating patients for clinician review.


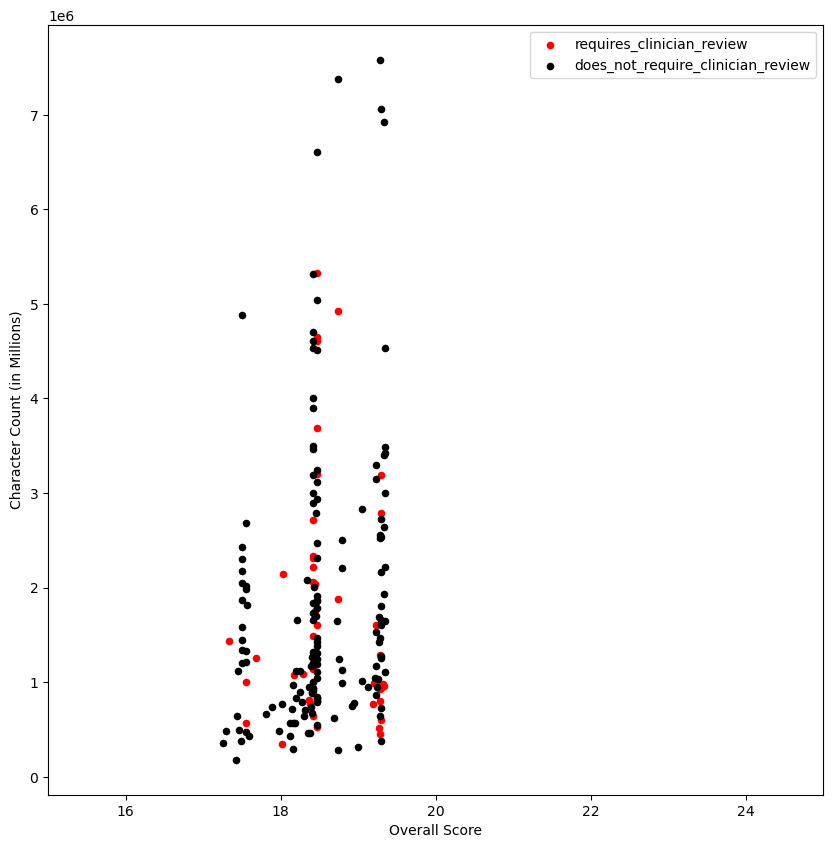


**Table S2.** Summary of manual annotations on 273 patients in the training data set.

| **Concept** | **Positive** | **Negated** | **NotPatient** | **Hypothetical** |
| --- | --- | --- | --- | --- |
| **Developmental_Delay** | **530** | **267** | **123** | **31** |
| **Insomnia** | **79** | **58** | **1** | **2** |
| **Epilepsy_or_Seizures** | **2030** | **771** | **579** | **750** |
| **Mood_Disturbance** | **321** | **65** | **78** | **13** |
| **Hypotonia** | **112** | **284** | **10** | **4** |
| **Movement_Disorders** | **52** | **184** | **12** | **23** |
| **Feeding_Issues** | **207** | **39** | **4** | **2** |
| **Oculogyric_Crisis** | **37** | **7** | **0** | **1** |
| **Cerebral_Palsy** | **89** | **6** | **8** | **2** |
| **Autonomic_Dysfunction** | **37** | **6** | **7** | **10** |
| **AADC_Deficiency** | **1** | **0** | **0** | **0** |

**
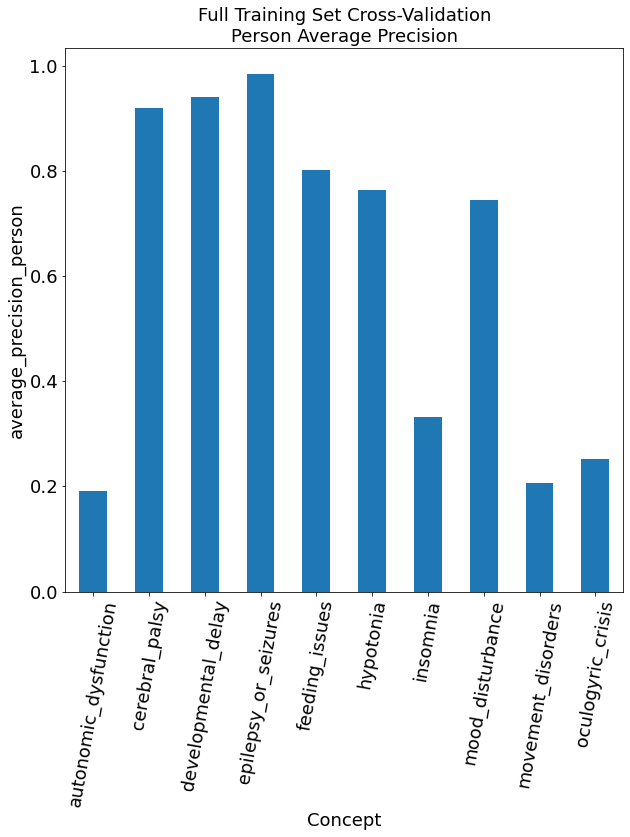
Figure S2.** Per-person average precision of the final concept models, computed by 5x 2-way cross-validation on the training data set.

**Figure S3.** Per-person average precision of the final concept models, computed on the validation data set.

**
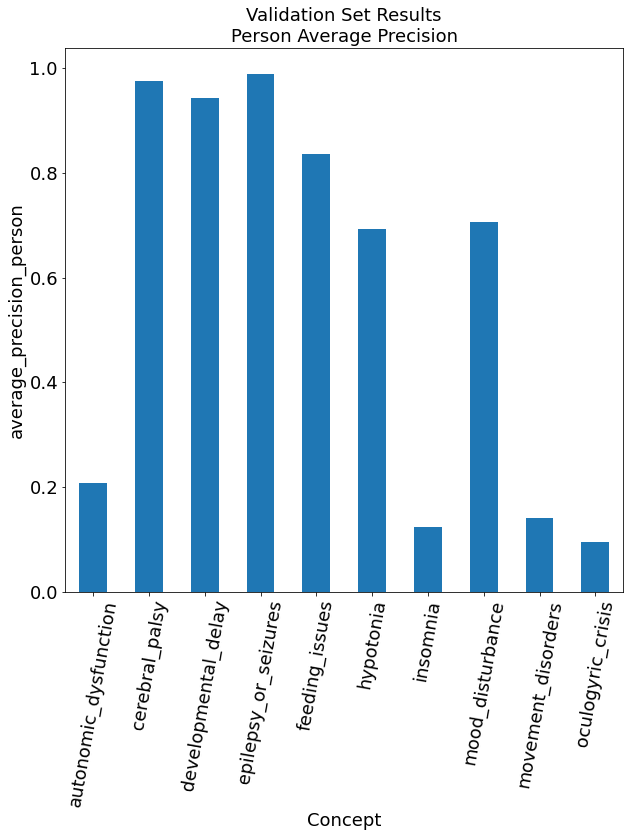
**

**Figure S4.** Per-person average precision of the final concept models, computed on the test data set.

**
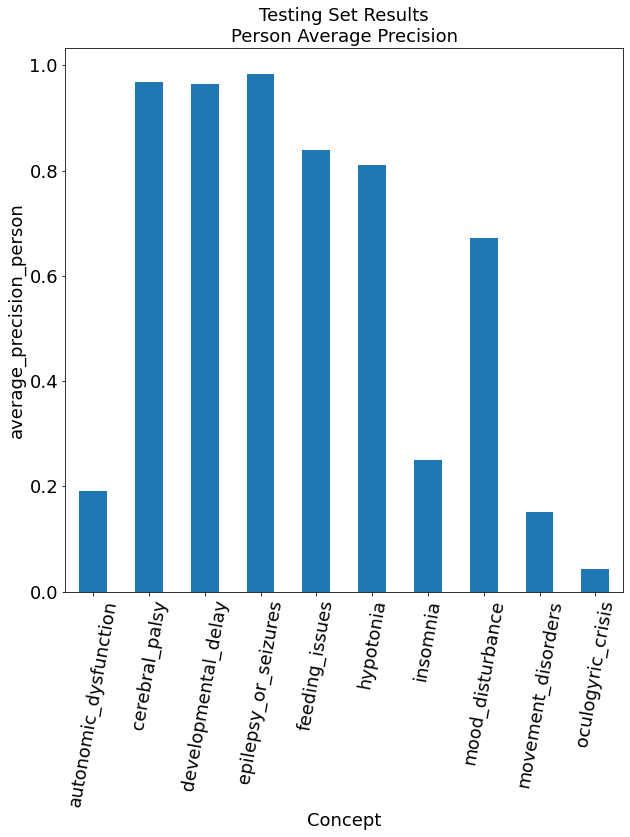
**

**Figure S5.** Per-person average precision of the final concept models, computed on the validation + test data set.

**
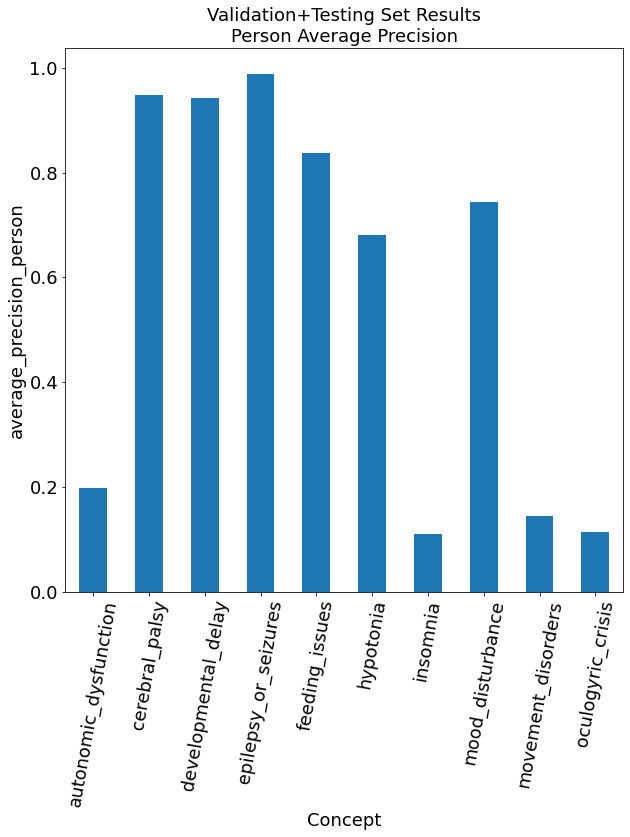
**

**Figure S6.** Distribution of Concept Probabilistic Predictions Person Positive (Green) vs. Negative (Red) on Validation Dataset.

**
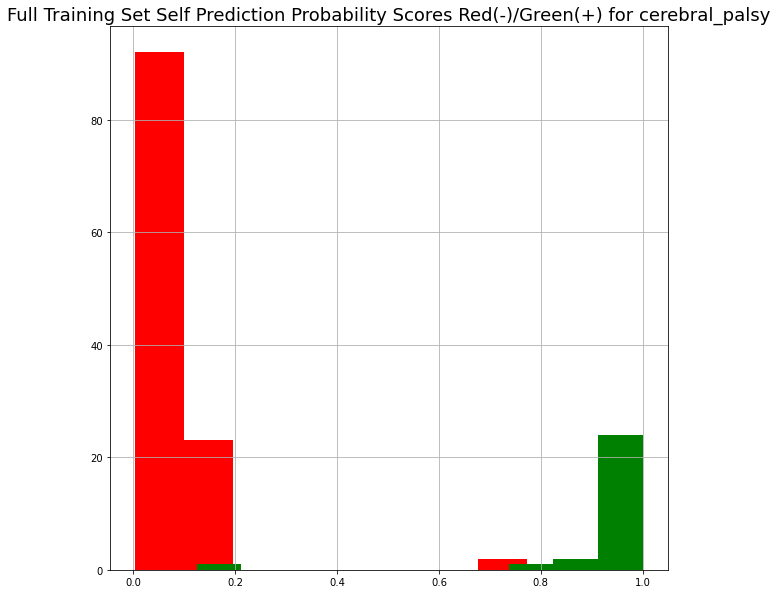
**

**
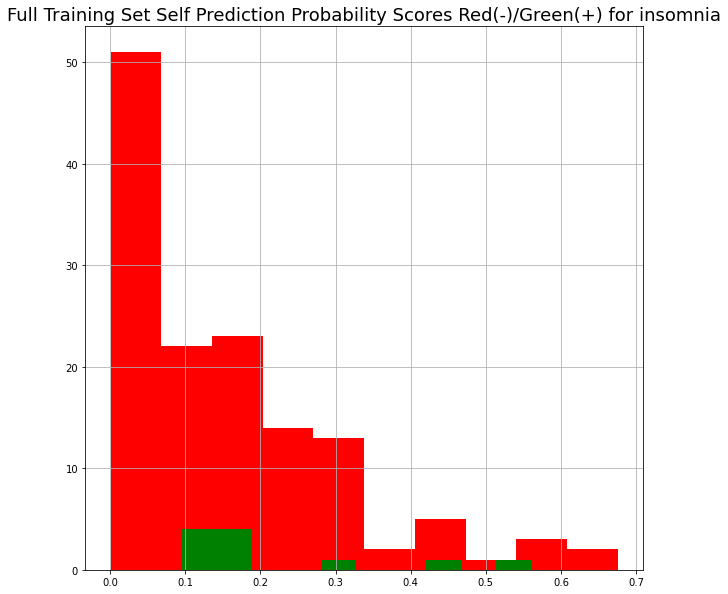

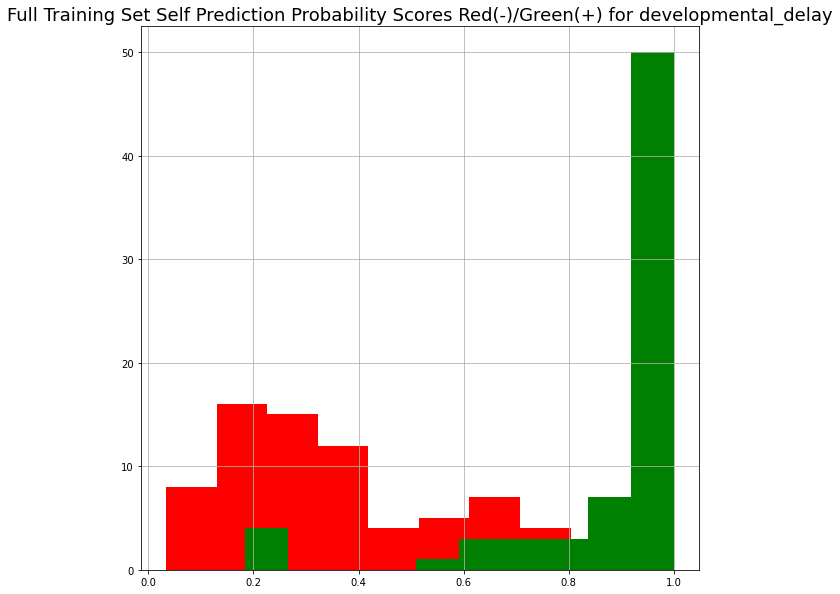

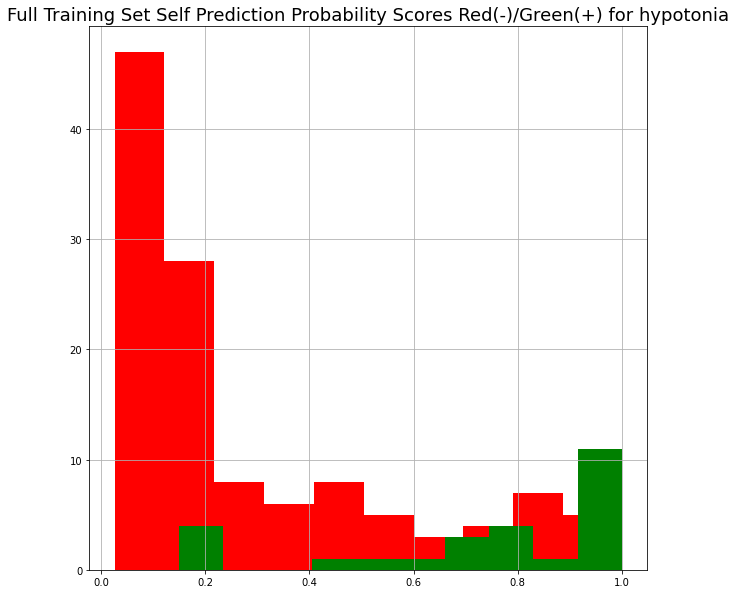
**

**
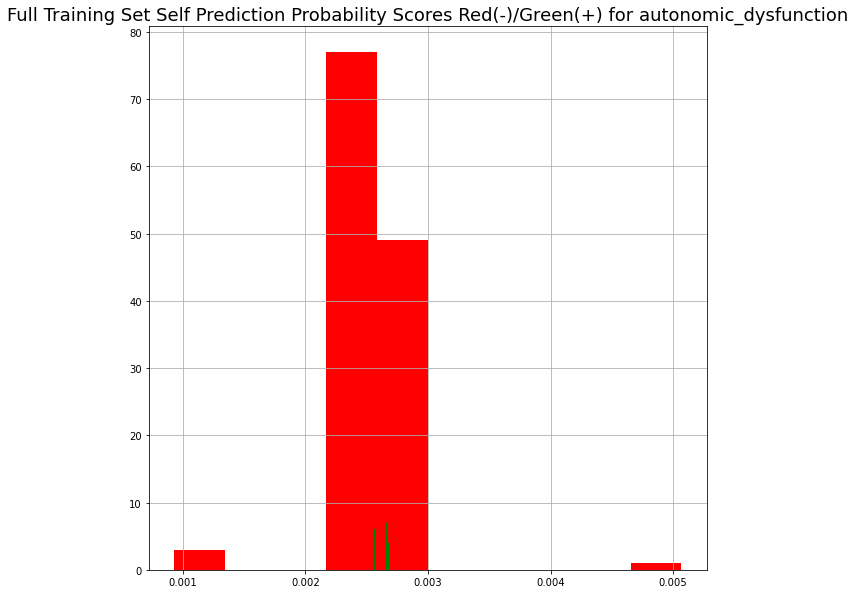

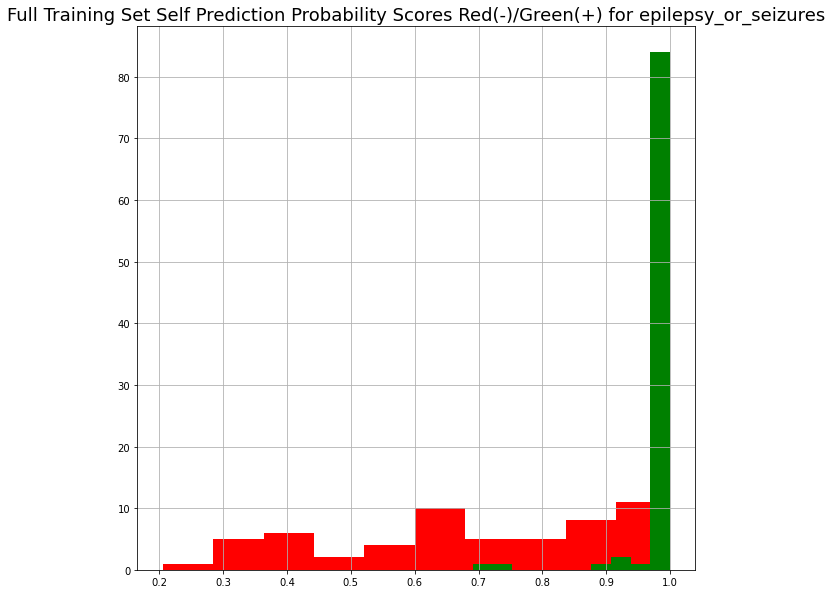

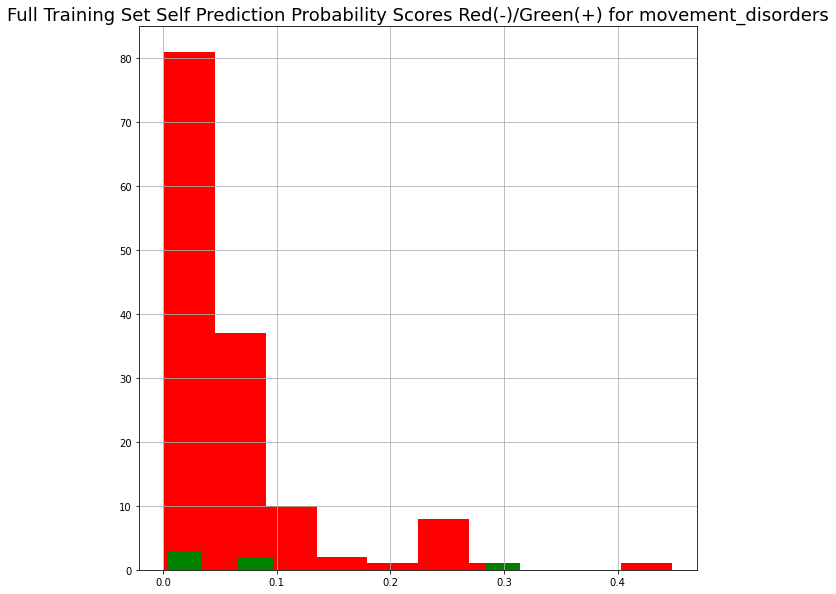

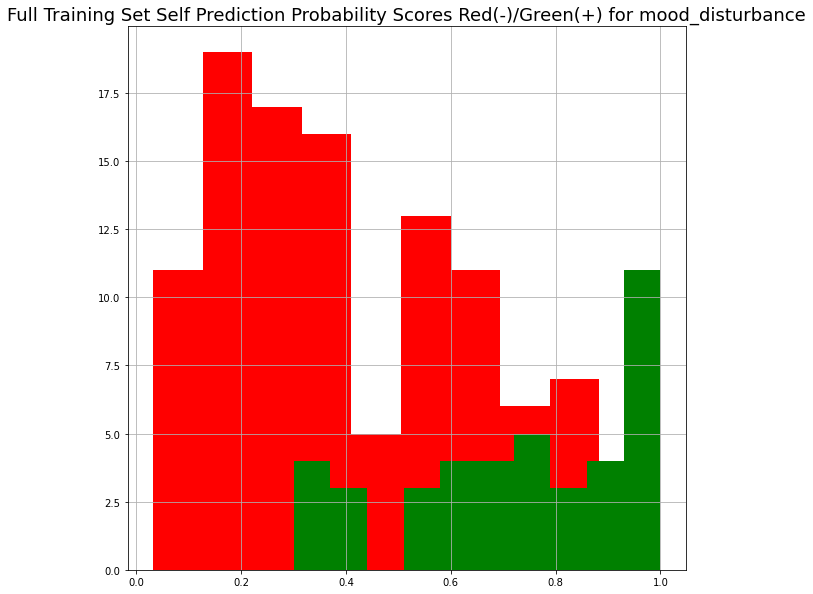
**

**Figure S7.** Comparison of reduction function average precision on the training data set using 5x 2-way cross-validation to compute the sentence level concept predictions.

**
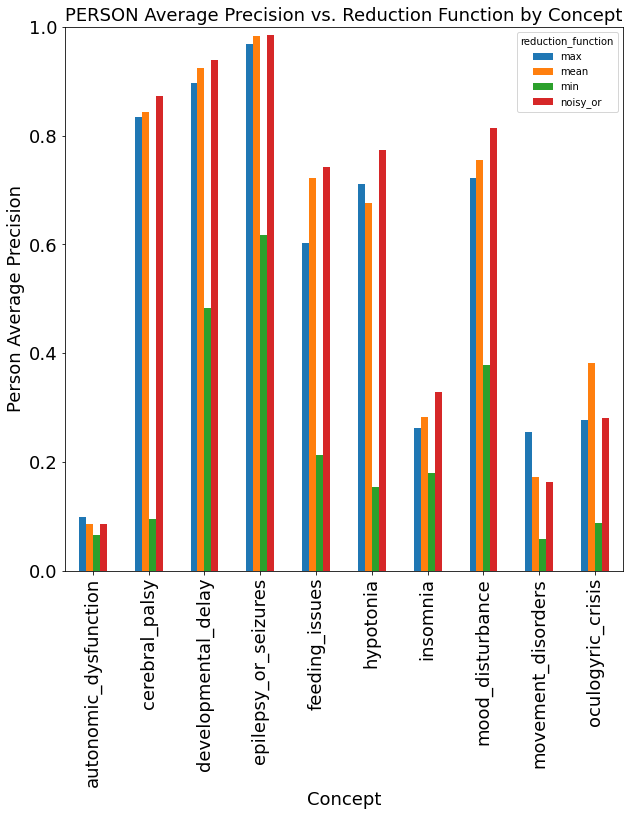
**

**Figure S8.** Negative log-loss performance comparison of parameter settings for the SVM linear kernel on all concepts. Similar plots were generated for the radial basis function, sigmoid, polynomial degree 2, and polynomial degree 3 kernels.

**
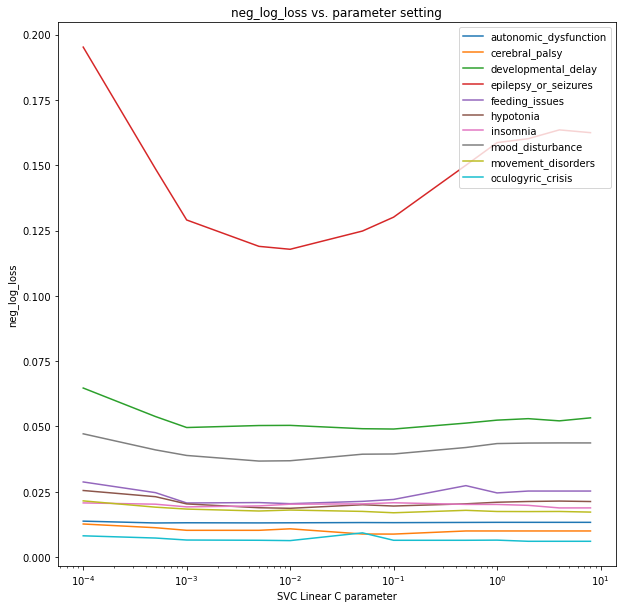
**

**Figure S8.** Distribution of patients across the 10 partitions which were divided randomly. Partition 0 was manually annotated and used for the training, validation, and test data sets in 80%/10%/10% proportion, Partitions 1-9 were not used until the final application of the algorithm and manual review of the top and bottom 200 ranked patients in this group of partitions.

**
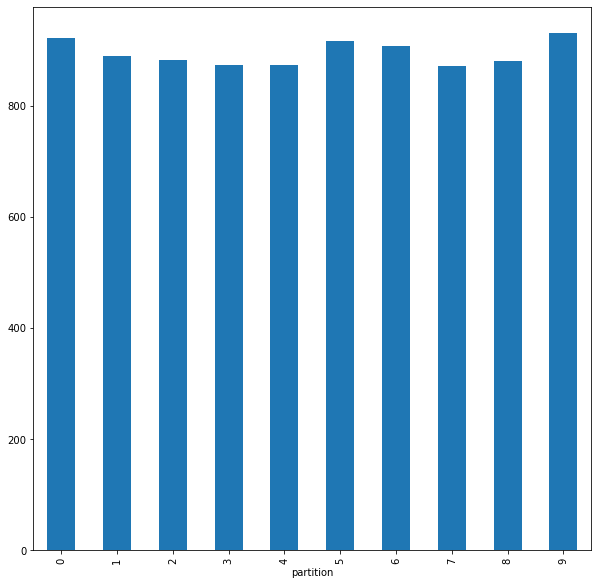
**

**Supplemental Appendix**

**Annotation Guide**

**AADC Concepts, Signs and Symptoms Annotation Guide**

Version 5.3

Last updated 4/21/2022

We will be using the BRAT annotation tool (https://brat.nlplab.org/) to highlight and mark text in clinical notes for concepts relevant to AADC diagnosis. For each concept mentioned in the medical record files provided to the annotator, the text stating or describing the concept should be highlighted and assigned on of the concepts from the concept set below. Concept text does not have to use the words or terms shown in the concept set if the annotator is confident that the words in the text express the concept under consideration. See the Concept Set Definitions and Indicators below as an aid. However, the annotatable text is not necessarily limited to that listed below.

If there are any relevant concept modifiers, these should be applied to the annotation as well using the BRAT modifier interface.

Annotations should be done to the finest level of granularity that encompasses complete concepts. A span should be annotated to the most specific concept. Also, each span should encompass a single concept, if possible, based on the grammatical structure of the sentence.

For example, if a text span describes **Hypotonia** the annotator should annotate this span with **Hypotonia** and not a more general term like **Movement Disorder**. If a sentence includes several concepts, each of these concepts should be annotated individually.

The BRAT interface is folder structured. Each patient is represented by their own folder. Within each patient folder there are folders representing each encounter. Within each encounter there are documents for clinical notes, as well as a document showing all the structured information for that encounter.

There is a special *patient_diagnosis* document at the top level of each patient folder that is used to annotate whether or not the patient has AADC Deficiency. This document has two lines in it:

Has AADC Deficiency

Does Not Have AADC Deficiency

After reviewing the patient folder, the annotator should annotate the patient_diagnosis document. If, in the clinical opinion of the annotator, the patient HAS AADC Deficiency the phrase “Has AADC Deficiency” should be selected and assigned the **Aromatic I-amino acid decarboxylase (AADC) deficiency** concept. If the patient DOES NOT HAVE AADC Deficiency the phrase “Does Not Have AADC Deficiency” should be selected and assigned the **Aromatic I-amino acid decarboxylase (AADC) deficiency** concept AS WELL AS THE **Negated** modifier.

**Concept Set**

1. Aromatic I-amino acid decarboxylase (AADC) deficiency
2. Oculogyric Crisis (OGC)
3. Hypotonia
4. Movement Disorders
5. Cerebral Palsy
6. Developmental Delay
7. Epilepsy/Seizures
8. Autonomic Dysfunction
9. Feeding Issues
10. Insomnia
11. Mood Disturbance

**Modifiers**

- (No modifier) The concept applies to the patient in a positive sense. The text is stating that the patient has the diagnosis or has experienced the symptom, either currently or in the past.
- **Negated**. The concept applies to the patient in a negative sense. The text is stating that the patient has NOT had the diagnosis or has experienced the symptom, either currently or in the past.
- **Hypothetical**. The text states that the patient may have had or experienced the concept, but is indecisive or uncertain.
- **NotPatient**. The text statement of the concept does not refer to the patient. It refers to background or general information or to a family member or relative or another person not the patient.

**Concept Set Definitions and Indicators**

| **Concept** | **Definition (from MeSH)** | **Synonyms and Keywords** |
| --- | --- | --- |
| Aromatic I-amino acid decarboxylase (AADC) deficiency | An autosomal recessive inborn error in neurotransmitter metabolism that leads to combined SEROTONIN and CATECHOLAMINE deficiency and is clinically characterized by vegetative symptoms, oculogyric crises, DYSTONIA, and severe neurologic dysfunction, usually beginning in infancy or childhood. Caused by mutations in the DDC gene. | Aadc  aromatic l-amino acid decarboxylase disorder, aromatic amino acid decarboxylase deficiency |
| Oculogyric Crisis (OGC) | acute [dystonia](https://www.merriam-webster.com/dictionary/dystonia#medicalDictionary) of the ocular muscles that is marked by involuntary intermittent or sustained deviation of the eyes in a usually upward direction and is often an adverse reaction to certain medications (as antipsychotic or antiemetic drugs) but may be associated with certain medical conditions (as postencephalitic parkinsonism). [https://www.merriam-webster.com/medical/oculogyric%20crisis]  Oculogyric crises are defined as spasmodic movements of the eyeballs into a fixed position, usually upwards. These episodes generally last minutes, but can range from seconds to hours.[^1^](https://www.ncbi.nlm.nih.gov/pmc/articles/PMC5623758/#b01) At the same time there is often increased blinking of the eyes and these episodes are frequently accompanied by pain. The patient can also experience neck dystonia and/or tongue protrusion. [https://dx.doi.org/10.7916%2FD85X2N2D] | oculogyric spasm, ogc,  oculogyric crisis,  oculogyric crises, orofacial dystonia, opisthotonic posturing, involuntary eye deviation(s), upward deviation of the eyes, ocular deviation(s),  abnormal eye movements,  ogc, persistent upgaze/eyes rolling back, intermittent eye rolling, eyes rolling back in a cluster |
| Hypotonia | A diminution of the skeletal muscle tone marked by a diminished resistance to passive stretching. | hypotonia, truncal hypotonia, floppy baby, muscle weakness, poor head control, head lag, ptosis  axial hypotonia, congenital hypotonia, low axial tone with peripheral hypertonia, muscle weakness (generalized), hypotonia in infancy, axial hypotonia with appendicular hypertonia (neonatal onset), pupillary changes |
| Movement Disorders | An attitude or posture due to the co-contraction of agonists and antagonist muscles in one region of the body. It most often affects the large axial muscles of the trunk and limb girdles. Conditions which feature persistent or recurrent episodes of dystonia as a primary manifestation of disease are referred to as DYSTONIC DISORDERS. | dystonia, limb dystonia, dystonic posturing, dyskinesia, hypokinesia, choreoathetosis, myoclonis, paroxysmal dystonia  movement disorders, spastic diplegia, parkinsonism |
| Cerebral Palsy | A heterogeneous group of nonprogressive motor disorders caused by chronic brain injuries that originate in the prenatal period, perinatal period, or first few years of life. The four major subtypes are spastic, athetoid, ataxic, and mixed **cerebral palsy**, with spastic forms being the most common. The motor disorder may range from difficulties with fine motor control to severe spasticity (see MUSCLE SPASTICITY) in all limbs. Spastic diplegia (Little disease) is the most common subtype, and is characterized by spasticity that is more prominent in the legs than in the arms. Pathologically, this condition may be associated with LEUKOMALACIA, PERIVENTRICULAR. | cerebral palsy  cp, mixed hypotonic/hypertonic cerebral palsy |
| Developmental Delay | Disorders in which there is a delay in development based on that expected for a given age level or stage of development. These impairments or disabilities originate before age 18, may be expected to continue indefinitely, and constitute a substantial impairment. Biological and nonbiological factors are involved in these disorders. | psychomotor delay,  delayed speech development,  delayed motor development,  delayed cognitive development,  Behavioral problems,  autistic features, global developmental delay, delayed milestones, gross motor delay  language development disorder, mixed receptive-expressive language disorder, intellectual disability, developmental deficits, developmental disability, learning disabilities |
| Epilepsy/Seizures | A disorder characterized by recurrent episodes of paroxysmal brain dysfunction due to a sudden, disorderly, and excessive neuronal discharge. Epilepsy classification systems are generally based upon: (1) clinical features of the seizure episodes (e.g., motor seizure), (2) etiology (e.g., post-traumatic), (3) anatomic site of seizure origin (e.g., frontal lobe seizure), (4) tendency to spread to other structures in the brain, and (5) temporal patterns (e.g., nocturnal epilepsy).  Seizures are Clinical or subclinical disturbances of cortical function due to a sudden, abnormal, excessive, and disorganized discharge of brain cells. Clinical manifestations include abnormal motor, sensory and psychic phenomena. Recurrent seizures are usually referred to as EPILEPSY or "seizure disorder." | seizure(s)  epilepsy  spell(s)    abnormal head movement(s)  spasm(s)  fit(s)  convulsion(s)  attack(s)  bout(s)  episode(s)  head turning  epileptic discharge  epileptiform discharge  spike waves  spike discharges |
| Autonomic Dysfunction | Diseases of the parasympathetic or sympathetic divisions of the AUTONOMIC NERVOUS SYSTEM; which has components located in the CENTRAL NERVOUS SYSTEM and PERIPHERAL NERVOUS SYSTEM. Autonomic dysfunction may be associated with HYPOTHALAMIC DISEASES; BRAIN STEM disorders; SPINAL CORD DISEASES; and PERIPHERAL NERVOUS SYSTEM DISEASES. Manifestations include impairments of vegetative functions including the maintenance of BLOOD PRESSURE; HEART RATE; pupil function; SWEATING; REPRODUCTIVE AND URINARY PHYSIOLOGY; and DIGESTION. | Autonomic dysfunction,  Autonomic abnormalities,  Hypoglycemia,  , temperature instability, excessive sweating (hyperhidrosis), diaphoresis, intermittent hypothermia, hypotension, dysautonomia, ptosis,  orthostatic hypotension, abnormal heart rhythm, arrhythmia, bradycardia, stridor, nasal congestion |
| Feeding Issues | A condition of substandard growth or diminished capacity to maintain normal function. | Failure to thrive, feeding problems, feeding difficulty, dysphagia, g-tube placement, g-tube dependence, poor feeding, constipation, reflux, diarrhea, excessive drooling, hypersalivation, dysmotility,  gjt dependent, feeding problem of newborn, constitutional failure to thrive, early feeding issues, oralpharyngeal dysphagia |
| Insomnia | Disorders characterized by impairment of the ability to initiate or maintain sleep. This may occur as a primary disorder or in association with another medical or psychiatric condition. | Sleep disturbances, sleepiness, insomnia, hypersomnia, abnormal sleep pattern  Sleep disorder, behavioral sleep insomnia of childhood, trouble sleeping |
| Mood disturbance |  | Irritability, dysphoria, excessive crying, emotional lability  Behavioral challenges, neurobehavioral difficulties, disruptive behavior disorder |

**References**

Pearson TS, Gilbert L, Opladen T, et al. AADC deficiency from infancy to adulthood: Symptoms and developmental outcome in an international cohort of 63 patients. *J Inherit Metab Dis*. 2020;43(5):1121-1130. doi:10.1002/jimd.12247

Wassenberg T, Molero-Luis M, Jeltsch K, et al. Consensus guideline for the diagnosis and treatment of aromatic l-amino acid decarboxylase (AADC) deficiency. *Orphanet J Rare Dis*. 2017;12(1):12. Published 2017 Jan 18. doi:10.1186/s13023-016-0522-z

Helman G, Pappa MB, Pearl PL. Widening Phenotypic Spectrum of AADC Deficiency, a Disorder of Dopamine and Serotonin Synthesis [published correction appears in JIMD Rep. 2014;17:97]. *JIMD Rep*. 2014;17:23-27. doi:10.1007/8904_2014_327

Dai W, Lu D, Gu X, Yu Y; Mainland Chinese League of AADC Rare Disease. Aromatic L-amino acid decarboxylase deficiency in 17 Mainland China patients: Clinical phenotype, molecular spectrum, and therapy overview. *Mol Genet Genomic Med*. 2020;8(3):e1143. doi:10.1002/mgg3.1143

Hwu WL, Chien YH, Lee NC, Li MH. Natural History of Aromatic L-Amino Acid Decarboxylase Deficiency in Taiwan. *JIMD Rep*. 2018;40:1-6. doi:10.1007/8904_2017_54

Pons R, Ford B, Chiriboga CA, et al. Aromatic L-amino acid decarboxylase deficiency: clinical features, treatment, and prognosis. *Neurology*. 2004;62(7):1058-1065. doi:10.1212/wnl.62.7.1058
